# A validated single-cell-based strategy to identify diagnostic and therapeutic targets in complex diseases

**DOI:** 10.1186/s13073-019-0657-3

**Published:** 2019-07-30

**Authors:** Danuta R. Gawel, Jordi Serra-Musach, Sandra Lilja, Jesper Aagesen, Alex Arenas, Bengt Asking, Malin Bengnér, Janne Björkander, Sophie Biggs, Jan Ernerudh, Henrik Hjortswang, Jan-Erik Karlsson, Mattias Köpsen, Eun Jung Lee, Antonio Lentini, Xinxiu Li, Mattias Magnusson, David Martínez-Enguita, Andreas Matussek, Colm E. Nestor, Samuel Schäfer, Oliver Seifert, Ceylan Sonmez, Henrik Stjernman, Andreas Tjärnberg, Simon Wu, Karin Åkesson, Alex K. Shalek, Margaretha Stenmarker, Huan Zhang, Mika Gustafsson, Mikael Benson

**Affiliations:** 1 Centre for Personalized Medicine, Linköping University, Linköping, Sweden; 2 Department of Internal Medicine, Region Jönköping County, Jönköping, Sweden; 3 Departament d’Enginyeria Informàtica i Matemàtiques, Universitat Rovira i Virgili, Tarragona, Spain; 4 Department of Surgery, Region Jönköping County, Jönköping, Sweden; 5 Office for Control of Communicable Diseases, Region Jönköping County, Jönköping, Sweden; 6 Division of Rheumatology, Autoimmunity, and Immune Regulation, Department of Clinical and Experimental Medicine, Linköping University, Linköping, Sweden; 7 Department of Clinical Immunology and Transfusion Medicine, Linköping University, Linköping, Sweden; 8 Department of Gastroenterology and Department of Clinical and Experimental Medicine, Linköping University, Linköping, Sweden; 9 Department of Medical and Health Sciences, Linköping University, Linköping, Sweden; 10 Bioinformatics, Department of Physics, Chemistry and Biology, Linköping University, Linköping, Sweden; 11 Department of Otorhinolaryngology, Yonsei University College of Medicine, Seoul, Korea; 12 Clinical Microbiology, Region Jönköping County, Jönköping, Sweden; 13 Division of Clinical Microbiology, Department of Laboratory Medicine, Karolinska Institute, Karolinska University Hospital Huddinge, Stockholm, Sweden; 14 Karolinska University Laboratory, Karolinska University Hospital, Solna, Sweden; 15 Department of Dermatology and Venereology, Region Jönköping County, Jönköping, Sweden; 16 Department of Clinical and Experimental Medicine, Faculty of Medicine and Health Sciences, Linköping University, Linköping, Sweden; 17 Futurum – Academy for Health and Care, Department of Pediatrics, Region Jönköping County, Jönköping, Sweden; 18 Institute for Medical Engineering and Science, Massachusetts Institute of Technology, Cambridge, MA USA; 19 Department of Chemistry, Massachusetts Institute of Technology, Cambridge, MA USA; 20 Koch Institute for Integrative Cancer Research, Massachusetts Institute of Technology, Cambridge, MA USA; 21 Broad Institute of MIT and Harvard, Cambridge, MA USA; 22 Ragon Institute of MGH, MIT and Harvard, Cambridge, MA USA; 23 Department of Pediatrics, Institution for Clinical Sciences, Göteborg, Sweden

**Keywords:** Network tools, scRNA-seq, Biomarker and drug discovery

## Abstract

**Background:**

Genomic medicine has paved the way for identifying biomarkers and therapeutically actionable targets for complex diseases, but is complicated by the involvement of thousands of variably expressed genes across multiple cell types. Single-cell RNA-sequencing study (scRNA-seq) allows the characterization of such complex changes in whole organs.

**Methods:**

The study is based on applying network tools to organize and analyze scRNA-seq data from a mouse model of arthritis and human rheumatoid arthritis, in order to find diagnostic biomarkers and therapeutic targets. Diagnostic validation studies were performed using expression profiling data and potential protein biomarkers from prospective clinical studies of 13 diseases. A candidate drug was examined by a treatment study of a mouse model of arthritis, using phenotypic, immunohistochemical, and cellular analyses as read-outs.

**Results:**

We performed the first systematic analysis of pathways, potential biomarkers, and drug targets in scRNA-seq data from a complex disease, starting with inflamed joints and lymph nodes from a mouse model of arthritis. We found the involvement of hundreds of pathways, biomarkers, and drug targets that differed greatly between cell types. Analyses of scRNA-seq and GWAS data from human rheumatoid arthritis (RA) supported a similar dispersion of pathogenic mechanisms in different cell types. Thus, systems-level approaches to prioritize biomarkers and drugs are needed. Here, we present a prioritization strategy that is based on constructing network models of disease-associated cell types and interactions using scRNA-seq data from our mouse model of arthritis, as well as human RA, which we term multicellular disease models (MCDMs). We find that the network centrality of MCDM cell types correlates with the enrichment of genes harboring genetic variants associated with RA and thus could potentially be used to prioritize cell types and genes for diagnostics and therapeutics. We validated this hypothesis in a large-scale study of patients with 13 different autoimmune, allergic, infectious, malignant, endocrine, metabolic, and cardiovascular diseases, as well as a therapeutic study of the mouse arthritis model.

**Conclusions:**

Overall, our results support that our strategy has the potential to help prioritize diagnostic and therapeutic targets in human disease.

**Electronic supplementary material:**

The online version of this article (10.1186/s13073-019-0657-3) contains supplementary material, which is available to authorized users.

## Background

One of the most important problems in health care today is that many patients do not respond to drug treatment. According to an FDA report, this affects 38–75% of patients with common diseases [1]. This problem is closely linked to increasing costs and difficulties in drug development [2]. One important driver of this high rate of failure is suggested by genome-wide association studies (GWAS), which identify increasing numbers of genetic variants that may affect highly diverse pathways and cell types in the same disease [3–14]. While genomic medicine has paved the way for addressing this diversity [15], the scale of the problem is indicated by single-cell RNA-sequencing (scRNA-seq) studies, which have shown altered expression of thousands of genes across many different cell types [16–23]*.* While such studies have resulted in the identification of potential novel disease mechanisms, no single-cell type, pathway, or gene has been shown to have a key regulatory role in any disease. Instead, the dispersion of multiple causal mechanisms across multiple cell types is supported by several other studies [6, 8, 9, 24]. An extreme consequence of such complexity could be that a prohibitive number of drugs may be needed for effective treatment of each disease. To address this problem, we would ideally need to (1) characterize all disease-associated cell types and pathways, followed by (2) prioritization of the relatively most important. To our knowledge, neither of these two challenges has been systematically addressed. One reason is that many cell types may not be accessible in patients, and another reason lack of methods to prioritize between the cell types and pathways [24].

Here, we hypothesized that a solution to systematically investigate multicellular pathogenesis and its diagnostic and therapeutic implications could be to use scRNA-seq data to construct models of disease-associated cell types, their expression profiles, and putative interactions. We will henceforth refer to such models as multicellular disease models (MCDMs). The importance of interactions in an MCDM lies in that they link the cell types into networks. As a simplified example, if the interactions were unidirectional, they could be traced to find upstream cell types and mechanisms for therapeutic targeting. However, biological interactions are often more complex. We therefore hypothesized that network tools could be used to prioritize cell types, mechanisms, and potential drug targets. In support, methods from network science have been applied to analyze genome-wide data from different diseases [25, 26]. We and others have used such methods to identify biomarkers and therapeutic targets based on bulk expression profiling data of individual cell types [12, 27, 28], as well as to develop a mathematical framework to rank network nodes [29]. A core concept is that the most interconnected nodes in a network tend to be most important. Indeed, a large body of evidence supports that such analyses can be formalized and used to find crucial nodes in a wide range of systems, ranging from proteins essential for cell survival to relevant web pages in a Google search [30, 31]. Because many cell types are not accessible from patients, we started with a mouse disease model. We focused on a mouse model of antigen-induced arthritis (AIA), because it allows potential analysis of all cells in the target organ, joints, and adjacent lymph nodes. We used our recently developed method for translational scRNA-seq [32]. The resulting MCDMs and complementary analyses of patients with RA and 174 other diseases supported multicellular pathogenesis of great complexity. Our analyses indicate that network analyses of the MCDMs can help to prioritize cell types and genes for diagnostics and therapeutics. General applicability of our strategy was supported by prospective diagnostic studies of 151 patients with 13 autoimmune, allergic, infectious, malignant, endocrine, metabolic, and cardiovascular diseases, as well as 53 age- and sex-matched controls. The therapeutic potential of the strategy was supported by network-based analyses of these diseases, as well as a study of the mouse model of arthritis. Taken together, our results support that our strategy may have the potential to prioritize therapeutic and diagnostic targets in complex diseases.

## Methods

### Study design

In summary, this study describes a scalable, step-wise strategy to construct MCDMs and exploit them for diagnostics and therapeutics. The strategy was validated by both clinical and experimental studies. The strategy is based on applying network tools to organize and analyze scRNA-seq data from a mouse model of arthritis and human rheumatoid arthritis. Diagnostic validation studies were performed using expression profiling data and potential protein biomarkers from prospective clinical studies of 13 diseases. A candidate drug was examined by a treatment study of a mouse model of arthritis, using phenotypic, immunohistochemical, and cellular analyses as read-outs.

### Mouse model of rheumatoid arthritis

In order to construct high-resolution multicellular disease models (MCDMs), we first performed scRNA-seq analysis in a mouse model of antigen-induced arthritis (AIA). This model is suitable for generating MCDMs in an in vivo setting because most disease-associated cell types and subsets can be potentially identified in the inflamed joint and adjacent lymph nodes. Arthritis was triggered in six anesthetized female 129/SvE mice (aged 8 to 20 weeks) on day 21 by intra-articular injection of 30 μg methylated bovine serum albumin (mBSA) in 20 μL, in the left knee joint after subcutaneous pre-sensitization with 100 μg mBSA in incomplete Freund’s adjuvant and 200 μg mBSA in complete Freund’s adjuvant two (day 7) and three (day 1) weeks earlier, respectively. The right knee joint was injected with 20 μL phosphate-buffered saline (PBS) as negative control. In some experiments, mBSA was injected in both joints to allow assessment of arthritis and scRNA-seq data from joint cells from the same arthritic animal. One week after intra-articular injection of mBSA (day 28), mice were sacrificed, and joints were either used for immunohistochemistry or scRNA-seq. For assessment of the degree of arthritis, joints were routinely fixed in 4% paraformaldehyde (PFA), decalcified in formic acid/sodium citrate buffer, embedded in paraffin, and cut into 4-μm-thick sagittal sections before hematoxylin and eosin (H&E) staining, as described in [33]. Specimens were examined in a blinded manner for pannus formation, cartilage and subchondral bone destruction, and synovial hypertrophy on an arbitrary scale, 0–3, as described in [33]. Arthritis frequency (score one or higher) and arthritis severity (median score) were calculated and compared to non-arthritic controls using the Mann-Whitney *U* test. All experimental procedures were performed according to the guidelines provided by the Swedish Animal Welfare Act and approved by the Ethical Committee for research on animals in Stockholm, Sweden (N271-14). For scRNA-seq experiments (see below), joints and lymph nodes from naïve (control) and arthritic mice draining the sites used for mBSA injection (axillary and popliteal) were isolated and single-cell suspensions were prepared by triturating the joint and lymph nodes and passing them gently through a 70-μm cell strainer. Red blood cells were lysed by adding RBC lysis solution (Sigma-Aldrich) according to the manufacturer’s instructions.

### Single-cell RNA-seq wet lab protocol

All scRNA-seq experiments were performed using the Seq-Well technique [32]. Briefly, single-cell suspensions prepared from cultured cells or tissue samples using standard techniques were counted and co-loaded with barcoded and functionalized oligo-dT beads (Chemgenes, Wilmington, MA, USA; cat. no. MACOSKO-2011-10) on microwell arrays synthesized as described in [32]. For each sample, 20,000 live cells were loaded per array, and libraries from three samples were pooled together for sequencing, resulting in a coverage of 6.6 reads per base. The microwell arrays were then covered with previously plasma-treated polycarbonate membranes, and the membranes were allowed to seal to the bead and cell co-loaded microwell arrays at 37 °C for 30 min. Next, cell lysis and hybridization were performed, followed by bead removal, reverse transcription, and whole transcriptome amplification. Libraries were prepared for each sample using the Nextera XT DNA Library Preparation Kit (Illumina, San Diego, CA, USA; cat. no. FC-131-1096) according to the manufacturer’s instructions. Libraries were sequenced using the NextSeq 500/550 system, and sequencing results were analyzed as described below.

### Validation of the single-cell RNA-seq analytical process

To verify our scRNA-seq setup, we mixed the two colorectal cancer (CRC) cell lines SW480 and HT29 before application to the single-cell array and sequenced them altogether according to the procedures described above. These two CRC cell lines (SW480 and HT29) were a kind gift from Xiaofeng Sun (Linkoping University). We showed that the cells from our previously mixed cell lines were correctly assigned to their corresponding cell lines, verifying our single-cell sequencing and clustering methods (Additional file 1: Figure S1). Quality check and clustering was performed as described below, using 26 colon cancer cell lines profiled with microarrays (GSE10843). Here, a low cutoff of 4000 unique transcripts per cell was added as a criterion to Seurat (Additional file 1: Figure S2). In total, 233 cells passed the quality criteria and were separated into two main clusters: SW480 and HT29, as expected. However, a subcluster of SW480, with a profile resembling that of the SW620 CRC cell line, was also identified (Additional file 1: Figure S1).

### Single-cell RNA-seq data processing

The single-cell data was processed into digital gene expression matrices following James Nemesh, McCarrol’s lab Drop-seq Core Computational Protocol (version 1.0.1, http://mccarrolllab.com) using bcl2fastq Conversion and Picard software. The indexed reference for alignment of the reads was generated from GRCh38 (April 2017, Ensembl) for human data (validation of the wet lab and cell type identification protocols; see the “Methods” section) and GRCm38 (June 2017, Ensembl) for mouse data using STAR software. Only primary alignments towards the reference genome were considered during downstream analyses, according to the mapping quality using STAR software. The quality of cells was assessed by having a minimum of 10,000 reads, 400 transcripts, and less than 20% mitochondrial genes per cell. Outliers were then removed based on an overestimation of transcripts count, due to the risk of duplicates in the library resulting in two or more cells sharing a cell barcode. This resulted in a total of 7086 and 1333 cells for the joint and lymph node data, respectively. The single-cell data was then normalized using Seurat [34] for further analysis. To reduce the noise within the data, *K*-nearest neighbor smoothing was applied for each tissue matrix separately, using a minimum *k* of 5 or, if more than 5000 cells were captured, ~ 0.1% of the total number of cells [35].

### Cell type identification

To cluster the cells and define the cell types, reference component analysis (RCA) was performed [18]. Each cell was projected against reference bulk expression profiling data, which were generated or derived from public databases, as described for each dataset below. The RCA references were prepared as described in the original paper [18]. Briefly, all genes with log10 (fold change) expression values greater or equal than 1 in any sample, relative to the median across all samples, were included. For each cell, we also saved the Pearson correlation *p* value from the RCA algorithm. Next, the reference component features were calculated, and data were clustered in a stepwise procedure. First, cells were clustered as described above. Second, those cells with non-significant *p* values (*p* > 0.05) were removed. Cells within each cluster that were significantly correlated (Benjamini-Hochberg adjusted *p* < 0.05) with their RCA-predicted cell type were identified accordingly, and cells with non-significant correlations were labeled as undetermined.

To construct the reference for the cell type identification in mouse experiments, we used the data from the study GSE10246. The resulting reference contained 5030 genes and 31 cell types/states (Additional file 2). *t*-SNE plots of joint and lymph node cells were created using MATLAB function *tsne()*, with perplexity parameter set to 40, based on the distance matrix obtained from RCA. Cells were colored according to the clusters identified with RCA. For clustering of the CRC cell lines, a reference was constructed using microarray data from GSE10843 and the same parameters as for the mouse reference. The resulting reference contained 2303 genes and 26 different cell lines (Additional file 2).

### Identification of differentially expressed genes

For single-cell data, differentially expressed genes (DEGs) were identified between each cell type and all other cell types, within each tissue separately, using Monocle (version 2.6.1) [36, 37]. A negative binomial distribution was used to define the dataset with a lowest detection limit of 0.5. Genes detected in at least three cells within a group were considered as expressed. Genes were considered as significantly differentially expressed if the *q* value < 0.05. For microarray data, DEGs were identified using the LIMMA R package.

### Pathway, biomarkers, and drug target enrichment analyses

Identification of pathways, biomarkers, and therapeutic targets was done with Ingenuity Pathway Analysis software [38].

### Network construction and centrality analysis

Our work included the different analysis of two kinds of cell-cell interaction networks, where one is based on potential spatial interactions and the other on predicted molecular interactions (see details below). The former was used as a proxy for molecular interactions, either because the cell types were not accessible from human patients or to prioritize cell types and tissues for scRNA-seq studies in animal models or human patients. We have made the resulting models of each 175 diseases available for such prioritization. The spatial interaction network models were created by empirical knowledge of which cells could potentially interact in the body. Second, using scRNA-seq data, we derived a refined network models, MCDMs, in which interactions were based on possible regulatory molecular interactions. These were inferred using Ingenuity upstream regulation analysis of the differentially expressed genes in each cell type. We validated the inferred interactions by another, recently described method, which is based on ligand-receptor interactions. However, both cases represent networks of possible physical interactions between cell types. The spatial network represented an undirected average network consisting of more nodes (45 cell types), while the latter resulted in a more sophisticated weighted and directed network of fewer nodes (e.g., six cell types for the joint MCDM from mouse AIA). There are many different methods to measure centrality. Given that we have no information about the kind of paths in the MCDM that will provide the best functional representation of the underlying chain of gene-regulatory processes, and that testing many different methods would involve risk of over-parametrization, we assumed a maximum entropy principle and used the less biased theoretical-information approach that for navigation is random walk centrality [39]. This metric is based on the average length of an average random walker moving at the network considering the weights, which has also been used by others in molecular biology. As our work aimed for using centrality as a generalizable concept, we also tested other metrics, such as the subgraph closeness centrality that is based on that the flow is concentrated to the closest paths. We found that although less significant similar results held for this metric as well. In support of centrality analyses to prioritize cell types, we found tumor cells to be most central in scRNA-seq data from colorectal cancer and significant correlations between centrality in scRNA-seq data from both mouse AIA and human RA.

### Construction of MCDMs

We constructed the MCDMs using scRNA-seq data from colorectal cancer, mouse AIA (sick or healthy lymph nodes and joints), and human RA synovium. The MCDMs showed genome-wide mRNA expression of each cell type as well as potential types and directions of intercellular interactions. For MCDM construction, we started by identifying cell-type-specific genes, i.e., DEGs in one cell type compared with all others, using the methods described above. Using those gene lists, MCDMs were constructed. First, the Ingenuity Pathway Analysis (IPA) software was queried for prediction of the upstream regulators of cell-type-specific DEGs for each cell type separately. Here, we focused on upstream regulators that were secreted or membrane-bound. Next, we searched for predicted upstream regulators among the DEGs of other cell types. If such an upstream regulator was found, an interaction was assumed between the cell types.

To systematically validate the MCDM cellular interactions derived from Ingenuity, we used the novel CellPhoneDB [40] framework. CellPhoneDB is a publicly available curated repository of ligands, receptors, and their interactions, which integrates a statistical tool for the inference of cell-cell communication networks from human single-cell transcriptomic data. Specifically, cell-type interactions between ligands and receptors from mouse RA and healthy joint MCDMs, and mouse RA lymph node MCDM were analyzed with CellPhoneDB, using the default parameters (the ligands and receptors included should be expressed in at least 10% of the cells for each cluster, and the cluster labels of every cell were randomly permuted 1000 times). As CellPhoneDB is developed for human scRNA-seq data, mouse genes were mapped to human orthologs using the BioMart database [41]. In total, 6203 (82.2%) and 4808 (87.4%) mouse genes from RA and healthy joint, respectively, could be mapped to humans. An interaction between two cell types was considered significant if the CellPhoneDB analysis predicted any interaction between the cell types with a significance score of *p* < 0.05.

### Cell centrality analysis

Cell centrality was determined by the random walk centrality and subgraph centrality [39]. For the scRNA-seq MCDM, directions were derived from the IPA upstream regulatory analysis, and weights were added to the interactions to include biological information for the computation of the coefficient. The weights were based on the number of cells of each cell type and its number of predicted upstream regulators (as described in the “Construction of MCDMs” section). The assumption behind these weights is that the chances of interactions between cell types are likely to increase with the number of cells and upstream regulators. Specifically, the weight for each interaction was derived by multiplying the number of cells by the number of predicted upstream regulators for two interacting cell types. Finally, the centrality of each cell was determined by the subgraph centrality using the normalized weighted adjacency matrix [42]. In order to validate that centralities were not biased by our choice of the Ingenuity database, we recomputed MCDMs and centrality measures using publicly available ligand-receptor interactions in mice [43] instead of Ingenuity.

### Centrality analysis of MCDMs from colorectal tumors

The hypothesis behind these analyses was that, since tumor cells have a causal role, they should also be more central than the surrounding immune and stromal cells. To test this hypothesis, processed FPKM (fragments per kilobase of transcript per million reads mapped) values for a scRNA-seq experiment of colon cancer (GSE81861) were downloaded from Gene Expression Omnibus [18]. T cells, B cells, and undefined cells were re-clustered using RCA, generating a novel reference expression profiling compendium. This consisted of 49 microarrays profiling 11 samples: B cell, CD4+, CD8+, monocytes, natural killer cells, naïve T cells, PBMC, Th1, Th17, Th2, and T regulatory (Treg) cells. Microarrays were processed as described below. RCA reference was prepared as described above, i.e., following instructions in [18]. Briefly, we included all genes with log10 (fold change) expression values above one in any sample, relative to the median across all samples. For each cell, we saved the correlation *p* value from the RCA algorithm. Next, we checked if all cells within each cluster were significantly correlated (BH adjusted *p* < 0.05) with the RCA-predicted cell type from reference data. All cells that did not fulfill this requirement were labeled as undetermined. In total, 579 cells were analyzed (Additional file 2). The DEGs were identified using Monocle and a truncated normal distribution (*tobit*) owing to its FPKM format, and the lowest detection limit was set at 0.1. The *estimateSizeFactors()* function was used to normalize for differences in mRNAs recovered across cells, and genes that were expressed in at least three cells were considered as present within a group. A tumor MCDM was constructed and centrality analyses were performed as described above (Additional file 1: Figure S3).

### Generation of an expression profiling reference compendium of immune cells

This compendium was used as a reference for classifying cell types in the scRNA-seq data from colorectal cancer and for deconvolution analyses of expression profiling data from CD4+ T cells. PBMCs were isolated from human peripheral blood using Lymphoprep (Axis-Shield Density Gradient Media, Oslo, Norway; cat. no. 1114545) according to the manufacturer’s instructions. Total RNA was extracted from one million PBMCs for microarray analysis. CD4+ T cells were isolated from two thirds of the remaining PBMCs using the CD4+ T Cell Isolation Kit, human (Miltenyi Biotec, Bergisch Gladbach, Germany; cat. no. 130-096-533) and LS Separation Columns (Miltenyi Biotec, Bergisch Gladbach, Germany; cat. no. 130-042-401) according to the manufacturer’s instructions. The negative fraction contained the CD4+ T cells. Total RNA was extracted from one million CD4+ T cells for microarray analysis. Remaining of the CD4+ T cells were incubated with anti-human CD4-FITC (Miltenyi Biotec, Bergisch Gladbach, Germany; cat. no. 130-092-358), anti-human CD127-PE (Becton, Dickinson, Franklin Lakes, NJ, USA; cat. no. 561028), anti-human CD183 (CXCR3)-PerCP/Cy5.5 (Biolegend, San Diego, CA, USA; cat. no. 353713), anti-human CD196 (CCR6)-PE/Cy7 (Biolegend, San Diego, CA, USA; cat. no. 353417), anti-human CD45RA-APC (Biolegend, San Diego, CA, USA; cat. no. 304111), anti-human CD25-APC/Cy7 (Biolegend, San Diego, CA, USA; cat. no. 302613), and anti-human CD194 (CCR4)-PE/Dazzle (Biolegend, San Diego, CA, USA; cat. no. 359419) for fluorescence-activated cell sorting (FACS) of naïve CD4+ T cells (only for counting, CD4+CD45RA+), Th1 (CD4+CXCR3+CCR6-CCR4-), Th2 (CD4+CXCR3-CCR6-CCR4+), Th17 (CD4+CXCR3-CCR6+CCR4+), and Treg (CD4+CD127lowCD25hi) cells. The remaining third of the PBMCs was used to isolate naïve CD4+ T cells using the Naïve CD4+ T Cell Isolation Kit II, human (Miltenyi Biotec, Bergisch Gladbach, Germany; cat. no. 130-094-131) and LS Separation Columns (Miltenyi Biotec, Bergisch Gladbach, Germany; cat. no. 130-042-401) according to the manufacturer’s instructions. The negative fraction contained the naïve CD4+ T cells (NT cells). Total RNA was extracted from NT cells for microarray analysis. The positive fraction from NT magnetic isolation was pooled with the positive fraction from CD4+ T cell magnetic isolation and incubated with anti-human CD3-Pacific Blue (Biolegend, San Diego, CA, USA; cat. no. 300418), anti-human CD4-FITC (Miltenyi Biotec, Bergisch Gladbach, Germany; cat. no. 130-092-358), anti-human CD56 (NCAM)-PE (Biolegend, San Diego, CA, USA; cat. no. 318305), anti-human CD19-PerCP/Cy5.5 (Biolegend, San Diego, CA, USA; cat. no. 302229), anti-human CD14-PE/Cy7 (Biolegend, San Diego, CA, USA; cat. no. 325617), and anti-human CD8-APC (Becton, Dickinson, Franklin Lakes, NJ, USA; cat. no. 555369) antibodies for FACS of CD4+ (only for counting, CD3+CD4+) and CD8+ (CD3+CD8+) T cells, natural killer cells (CD56+), B cells (CD19+), and monocytes (SSClowCD14+). Total RNA was isolated from 11 cell types (PBMCs, B cells, CD4+ and CD8+ T cells, monocytes, natural killer cells, naïve T cells, Th1, Th17, Th2, and Treg cells using the AllPrep DNA/RNA Micro kit (Qiagen, Hilden, Germany; cat. no. 80284) according to the manufacturer’s instructions and used for microarray analysis.

### Cell centrality correlation with enrichment of genes harboring RA-associated genetic variants

Pearson correlation was calculated between subgraph centrality score and −log(*p* value) of the enrichment of genes harboring RA-associated genetic variants among the DEGs in each cell type. Genes harboring genetic variants associated with RA were downloaded from DisGeNet (February 2017), one of the largest publicly available collections of genes and genetic variants associated with human diseases (http://www.disgenet.org) [44]. We removed two long non-coding RNA, 21 gene symbols beginning with LOC, and three microRNAs, leaving 207 genes. Since the RA-gene associations were identified in human samples and RA cells were derived from a mouse model, we searched for mouse orthologs for all human RA-associated genes. The list of human and mouse orthologs was downloaded from Ensembl Compara in June 2017 (Ensembl version 89). For 169 out of 207 human RA associated genes, we identified mouse orthologs.

### Enrichment of human RA-associated genes in mouse MCDMs

Since RA-gene associations were identified in human samples and RA cells were derived from a mouse model, we used mouse orthologs for all genes harboring genetic variants associated with RA. The 169 mouse orthologs were used for enrichment (Additional file 2). As a background, we used all mouse genes annotated in the NCBI database on June 16, 2017. Enrichment results are reported in (Additional file 3: Tables S1 and S2).

### Identification of disease-associated genes and cell types by meta-analysis of genome-wide association studies and cell-type-specific epigenetic markers

We first identified diseases analyzed with genome-wide association studies (GWAS) by downloading GWAS data compiled by the National Human Genome Research Institute (NHGRI). First, we manually classified 180 traits as diseases (Additional file 2) and excluded the remaining traits from the analysis. The identified diseases belonged to 20 out of 21 disease chapters listed in ICD-10-CM (International Classification of Diseases, Tenth Revision, Clinical Modification, Additional file 2, Additional file 3: Table S3): infectious (I); neoplasms (II); diseases of the blood and blood-forming organs including immune mechanisms (III); endocrine, nutritional, and metabolic diseases (IV); mental and behavioral disorders (V); diseases of the nervous system (VI); diseases of the eye (VII); diseases of the ear (VIII); diseases of the circulatory system (IX); diseases of the respiratory system (X); diseases of the digestive system (XI); diseases of the skin (XII); diseases of the musculoskeletal system and connective tissue (XIII); diseases of the genitourinary system (XIV); pregnancy, childbirth and the puerperium (XV); conditions originating in the perinatal period (XVI); congenital malformations, deformations, and chromosomal abnormalities (XVII); symptoms, signs, and abnormal clinical and laboratory findings, not elsewhere classified (XVIII); injury, poisoning, and certain other consequences of external causes (XIX); and factors influencing health status and contact with health services (XXI). One hundred seventy-five of the diseases belonged to all 17 disease-associated chapters, while five diseases belonged to chapters XVIII, XIX, and XXI. The number of diseases in each ICD-10-CM chapter is illustrated in (Additional file 1: Figure S4).

Next, we downloaded single nucleotide polymorphisms (SNPs) associated with these diseases from DisGeNet (February 2017) [44], which integrates 46,589 unique SNPs from GWAS, expert-curated repositories, and scientific literature. In total, 9880 out of 46,589 SNPs (21.2%) were associated with the given 180 diseases (Additional file 2). Among these, 8316 SNPs were mapped to 4475 unique genes (3518 with Entrez identifier).

In order to identify cell types significantly associated with GWAS diseases, we selected cell types with cell-type-specific epigenetic markers significantly enriched for SNPs associated with each disease (Additional file 2). These cell types and their disease associations were compiled into a compendium, which will henceforth be referred to as CellComp (Additional file 1: Figure S5). First, we downloaded the processed BED files from ENCODE (https://www.encodeproject.org) for each of the cell types corresponding to healthy cells, which in total contained 45 cell types. We focused on nine epigenetic markers that are present in most cell types (Additional file 2). These cell types included those of the nervous, immune, and circulatory systems, as well as stromal and tissue-specific cell types.

GWAS disease SNPs overlapping with epigenetic markers were used to calculate a disease-cell type *p* value for each marker-disease-cell type triplet, using the Fisher exact test. Specifically, each disease was defined by the SNPs from DisGeNet (see above) in combination with all linkage disequilibrium (LD) SNPs associated with the disease SNP set, obtaining 175 out of the 180 diseases considered. LD SNPs were retrieved from SNAP (https://www.broadinstitute.org/snap/snap) using the R package “rsnps” with default parameters of *R*^2^ = 0.8, within 500 base pairs for each SNP. For each cell type and disease pair, we calculated an overlap Fisher exact *p* value. For each marker, we then formed a disease-disease similarity score based on similarities in their epigenetic associations by Pearson correlation of −log *p* values of the disease-epigenetic profiles, which resulted in a *p* value for the disease-disease associations for each disease pair and each marker. We then computed the direct genetic overlaps of all diseases and found them to be highly significant, although the marker with the lowest *p* value was H3K36me3 (Additional file 3: Table S4). For robustness analysis, we also computed a binomial enrichment test by counting the numbers of significant disease-cell associations (Fisher’s exact test, *p* < 0.05), which also showed significant enrichment for many markers. We performed the analysis by constructing a single *epigenetic disease association score* for each of the 45 cell types and 175 diseases by combining each of the *p* values from the markers using the Fisher method (also known as Fisher’s combined probability test method). Using this score, we found a significant association (Fisher’s exact test, *p* < 0.05) of each disease with a median range of 20 (0–45) cell types (Additional file 3: Table S4). The disease-cell associations are shown in a cluster diagram (Additional file 1: Figure S6).

### Pathway analysis of genes harboring disease-associated genetic variants

In order to identify the potentially most disease-relevant cell types in the MCDMs, we performed pathway analysis of the genes harboring genetic variants associated with the GWAS diseases, using the IPA software (Additional file 1: Figure S7). We found that the most significant pathways were immune-related, including the top scoring Th1 and Th2 activation pathway (*p* = 3.22 × 10^−34^). To examine if this result was caused by an overrepresentation of immune-related GWAS diseases, a medical doctor manually classified the diseases as either primarily immune or non-immune (Additional file 2). Then, we repeated the analyses for all primary non-immune-related diseases. This also resulted in the identification of the Th1 and Th2 activation pathway as one of the top scoring pathways (*p* = 3.33 × 10^−14^).

### Construction of a cell type-disease network

To get an overview of the disease-associated cell types, we constructed a network where cell types were depicted as nodes with sizes proportional to the number of diseases they were associated with. The nodes were linked based on manual curation of potential spatial interactions in the body. For example, bronchial epithelial cells can spatially interact with T lymphocytes but not with uroepithelial cells (Additional file 2).

In the resulting network, immune cells were most interconnected and also appeared to be associated with more diseases than less connected cells. Indeed, we found a significant positive correlation between the degree of the cell type and the number of diseases a cell type was associated with (from the e*pigenetic association score*, Pearson *r* = 0.31, *p* = 0.038). Moreover, we classified all cell types into cell categories, namely immune cells, epithelial cells, muscle cells, neural cells, hepatocytes, fibroblasts, and osteoblasts (Additional file 2). For each cell category, we calculated a general cell-class-disease association *p* value by Fisher combining *p* values for all diseases and all cell types in cell category. Small *p* values from the chi-square distribution were numerically approximated through a normal distribution approximation followed by Taylor series expansion of the cumulative probability distribution [35].

### Public profiling data of CD14+, CD4+, and B cells from patients with rheumatoid arthritis

For the construction of RA modules based on expression profiles in CD14+, CD4+ T cells, and B cells, we downloaded public microarray experiments from GEO. We analyzed the datasets GSE57386 (CD14+), GSE56649 (CD4+ T cells), and GSE4588 (B cells). Module construction and classification with elastic net were performed as described below.

### Prospective clinical expression profiling studies of CD4+ T cells from patients with 13 different diseases

We conducted prospective clinical studies to validate the importance of CD4+ T cells in 13 diseases from the following ICD-10-CM chapters: neoplasms (breast cancer, chronic lymphocytic leukemia); endocrine, nutritional, and metabolic diseases (type I diabetes, obesity); diseases of the circulatory system (atherosclerosis); diseases of the respiratory system (acute tonsillitis, influenza, seasonal allergic rhinitis, asthma); diseases of the digestive system (Crohn’s disease, ulcerative colitis); and diseases of the skin and subcutaneous tissue (atopic eczema, psoriatic disease).

Study participants were recruited by clinical specialists based on diagnostic criteria defined by organizations representing each specialist’s discipline. Age- and gender-matched healthy controls (*n* = 151 and 53, respectively) were recruited in the Southeast region of Sweden from outpatient clinics at the University Hospital, Linköping; Ryhov County Hospital, Jönköping, a primary healthcare center in Jönköping; and a medical specialist unit for children in Värnamo. Study participants represented both urban and rural populations with an age range of 8–94 years. Patients with type I diabetes and obesity had an age range of 8–18 years. Eleven patients had more than one diagnosis and are included in the reported patient numbers in the following description. For the bioinformatic analyses, when comparing patients with different diagnoses, patients suffering from both diseases in question were excluded (for example, when classifying patients with atherosclerosis versus influenza, patients having both of those diseases were excluded from this specific calculation).

#### ICD-10-CM chapter II: neoplasms

##### Breast cancer

Patients with breast cancer were recruited at first diagnosis at an outpatient clinic based on clinical examination (palpation), radiological analyses (mammography and ultrasonography), and pathologist’s evaluation of biopsy material from mastectomy and sentinel nodes. Blood sampling was performed before surgery, and all included patients had invasive ductal or lobular cancers. First, eight patients were recruited (median age [range], 73.5 [67–82] years). For a second validation study, an independent group of 24 patients (median age [range], 61.5 [35–88] years) was recruited, based on the same inclusion criteria. All recruited patients were women.

##### Chronic lymphocytic leukemia (CLL)

Patients (*n* = 8; three women; median age [range], 69.5 [51–80] years) with untreated CLL were recruited from two hematological outpatient clinics.

#### ICD-10-CM chapter IV: endocrine, nutritional, and metabolic diseases

##### Type I diabetes mellitus

Children and adolescents who met the criteria defined by the International Society for Pediatric and Adolescent Diabetes [8] were recruited, i.e., fast-plasma-glucose level above 7.0 mmol/L at two occasions, alternative non-fasting plasma glucose above 11.1 mmol/L, and symptoms of hyperglycemia. Patients with type I diabetes or those who received insulin treatment for more than 4 weeks were excluded (*n* = 8, two females; median age [range], 12.5 [11–16] years).

##### Obesity

Children who fulfilled international criteria for overweight or obesity were included based on standards for anthropometric measuring and those with diabetes mellitus were excluded [9]. Age- and gender-correlated body mass index (BMI) was calculated and defined as weight (kg) divided by stature square (m^2^). The median BMI was 28.0 (23.0–39.5). In total, 17 patients were recruited, including children (seven females; median age [range], 14.0 [8–60] years).

#### ICD-10-CM chapter IX: diseases of the circulatory system

##### Atherosclerosis

Patients were recruited by the same surgeon based on standard criteria [45] at an outpatient clinic, at least 3 months after coronary artery bypass graft surgery. In total, 12 patients were recruited (two females; median age [range], 71 [49–80] years). The patients were on continuous medication with statins. Patients with diabetes mellitus were excluded.

#### ICD-10-CM chapter X: diseases of the respiratory system

##### Acute tonsillitis

Patients (*n* = 6, six females; median age [range], 37.0 [26–46] years) with clinical signs of acute tonsillitis were recruited, and the diagnosis was confirmed through a rapid antigen diagnostic test or throat culture before the administration of antibiotics (*n =* 6).

##### Influenza

Patients with influenza A (*n* = 9) and influenza B (*n* = 1) were included in the study. Influenza diagnosis was verified by PCR analysis on nasopharyngeal secretions using the Xpert Flu/RSV XC assay (Cepheid, Sunnyvale, CA) according to the manufacturer’s instructions. A total number of 10 patients were recruited (four females; median age [range], 63.0 [23–97] years). Blood samples were drawn while the patients were still symptomatic. Most patients had not started any antiviral therapy at the time of sampling, but some had received one dose of oseltamivir.

##### Seasonal allergic rhinitis

In total, 13 patients with seasonal allergic rhinitis were recruited (11 females; median age [range], 38.0 [19–53] years) based on clinical history for at least two pollen seasons, and positive skin prick tests or radioallergosorbent tests (RAST) for birch or grass. Samples were obtained during the pollen season after at least 1 day of symptoms and before treatment.

##### Asthma

Patients were recruited based on standard criteria, i.e., at least 2-year history of recurrent wheezing and baseline bronchodilator reversibility of ≥ 12%. All patients were treated with inhaled glucocorticoids and bronchodilators as required. In total, 17 patients were recruited (six females; median age [range], 49.0 [16–74] years).

#### ICD-10-CM chapter XI: diseases of the digestive system

All patients with the inflammatory bowel diseases (IBDs) UC and CD were recruited at a gastroenterology outpatient clinic by the same gastroenterologist, based on clinical evaluation, endoscopy, and/or MRI, as well as characteristic histopathological findings and exclusion of differential diagnosis. 

##### Ulcerative colitis

In total, 10 patients with UC (five females; median 515 age [range], 51.5 [20–69] years were recruited.

##### Crohn´s disease

In total, 11 patients with CD (nine female; median age [range], 516 50.0 [31–76] years) were enrolled in the study.

All patients were in remission. None of the study subjects had received any systemic immunosuppressive medication three months prior to study entry.

#### ICD-10-CM chapter XII: diseases of the skin and subcutaneous tissue

All atopic eczema and psoriasis patients were diagnosed by the same dermatologist (OS), based on standard criteria, medical history, and/or histopathological findings, at a dermatology outpatient clinic.

##### Atopic eczema

In total, nine patients (three females; median age [range], 42.0 [12–76] years) were recruited.

##### Psoriasis

In total, 11 patients (six females; median age [range], 48.0 [20–71] years) with mild to severe plaque-type psoriasis were recruited. Atopic eczema patients had active eczema for at least 1 week, and a diagnosis for at least 2 years. Psoriasis patients were diagnosed for at least 1 year, and assessment of disease severity was performed using the Psoriasis Area and Severity Index (PASI). Median PASI was 8.1 (range, 4.2–16.2). None of the study subjects had received any systemic immunosuppressive medication or phototherapy 3 months prior to study entry.

### Isolation of peripheral CD4+ T cells

Briefly, PBMCs were prepared from fresh blood samples from the patients of 13 diseases and healthy controls, as previously described [12], using Lymphoprep (Axis-Shield PoC) according to the manufacturer’s protocol. Total CD4+ T cells were enriched from PBMCs by FACS. Human IgG (Sigma-Aldrich, St Louis, MO, USA) at a final concentration of 200 μg/mL was used to block cells prior to staining. Mouse anti-human CD4-FITC (BD Pharmingen San Diego, CA, USA), Mouse antihuman CD3-Pacific Blue (Biolegend San Diego, CA, USA), and all matched isotype controls were purchased. Cell sorting was performed on a FACS Aria flow cytometer (BD Biosciences, San Diego, CA, USA), and the data was analyzed by FlowJo 7.6 (Tree Star, Inc., San Carlos, CA). After sorting, the purity of total CD4+ T cells was more than 98%.

### Preparation of RNA for expression profiling

Total RNA was extracted using the AllPrep DNA/RNA Micro kit (Qiagen, Hilden, Germany; cat. no. 80284) according to the manufacturer’s instructions. RNA concentration and integrity were evaluated using the Agilent RNA 6000 Nano Kit (Agilent Technologies, Santa Clara, CA, USA; cat. no. 5067-1511) on an Agilent 2100 Bioanalyzer (Agilent Technologies, Santa Clara, CA, USA). Microarrays were then further computationally processed as described in One-Color Microarray-Based Gene Expression Analysis Low Input Quick Amp Labeling protocol (Agilent Technologies, Santa Clara, CA, USA).

### Microarray data processing

All gene expression microarrays were processed as described above using the LIMMA R package. All probes with an expression below 1.2 times the background signal were removed. To test whether the sex or age of the patients had any confounding effects on the CD4+ T cell microarray data for the 13 diseases, a principal component analysis (PCA) was performed. The results showed no clear differences in any of the components (Additional file 1: Figure S8). To test for possible confounding effects within T cell subsets, we performed deconvolution analysis of expression profiles from the CD4+ T cells using profiles from Th1, Th2, Th17, and Treg cells. Those profiles were derived from the above-described reference expression profiling compendium of human immune cells.

### Deconvolution of bulk CD4+ T cell data with sorted T cell subsets using CIBERSORT

We tested whether T cell subtypes differed significantly between sexes, ages, and diseases. For this purpose, we performed in vivo sorting of nine different immune cell types, where four were tested in this study, namely Th1, Th2, Th17, and Treg cells, followed by microarray analysis. Next, we applied CIBERSORT [46] with default parameters using our own reference transcriptomics data for each of the patients from our 13 diseases and the controls. This showed a high overlap of the different age groups, sexes, and diseases (Additional file 1: Figure S9).

### Human protein interactome

STRING (v.10) [47] was used to construct the human protein-protein interaction network (PPIn) as a representation of the human protein interactome. All the interactions with a confidence score greater than 0.7 were considered, for both direct (physical) and indirect (functional) interactions. The resulted network consisted of 11,228 vertices (proteins) and 212,419 edges (interactions).

### Module construction

The module construction was based on the integration of expression data for diseases and controls, and the PPIn. Given a disease *d*_*i*_, its associated module *M*_*i*_ was defined by the set of genes with highly correlated expression patterns, forming cliques into the PPIn and enriched for DEGs. Given the modules *M*_1_, ... , *M*_13_, the shared interaction neighborhood [9] was defined as the union of modules, comprising all the genes from all the disease modules. The modules consisted of median 392 genes [201–735]. A full list of individual module genes is provided in Additional file 2. Top canonical pathways enriched in module genes are reported in Additional file 2.

### Diagnostic potential of CD4+ T cell expression profiles

Classification of patients and controls was performed using elastic net function *lassoglm()* on MATLAB in the Statistics Toolbox, choosing lambda (*λ)* from the minimum deviance of leave-one-out cross-validation starting from all the measured genes of the platform. Prediction *p* values of case versus controls were based on the leave-one-out estimates. We calculated the area under the precision recall curve using the *perfcurve()* MATLAB function. The *p* values were calculated using the two-sided Wilcoxon rank-sum testing on the classifier discriminant function outputs.

For each of the 13 diseases, expression of disease-specific module genes separated patients from controls with high accuracy (median AUC 0.98, range 0.82–1; median *p* < 2.8 × 10^−5^, range 8.5 × 10^−8^ to 7.8 × 10^−4^). For example, the AUC for breast cancer was 1 (*p* = 1 × 10^−5^). An independent validation study of the module in 24 breast cancer patients and 14 healthy controls yielded an AUC of 0.82; *p* = 1.7 × 10^−3^, which was significantly higher than that for random genes (one-sided, permutation test, *p* < 3.7 × 10^−258^). We also found that the respective module union genes separated patients with different diagnoses from each other (median AUC 0.98, range 0.27–1; median *p* < 1.0 × 10^−3^, range 1.32 × 10^−5^ to 0.69; Additional file 2). Box plot was created using MATLAB *boxplot()* function with default settings. Outliers were defined by the algorithm underlying *boxplot()* function, i.e., points were assumed to be outliers if they were greater than *q*_3_ + *w* × (*q*_3_ − *q*_1_) or less than *q*_1_ − *w* × (*q*_3_ − *q*_1_), where *q*_*1*_ is the 25th percentile, *q*_*3*_ is the 75th percentile, and *w* corresponds to ± 2.7*σ* and 99.3% coverage according to the function description.

### Classification robustness was confirmed via 20 additional classifiers

Classification robustness was confirmed via 20 additional classifiers, namely, Coarse KNN, Cosine KNN, Fine KNN, Cubic KNN, Weighted KNN, Medium KNN, Complex Tree, Medium Tree, Simple Tree, Linear Discriminant, Logistic Regression, SVM Coarse Gaussian, SVM Cubic, SVM Fine Gaussian, SVM Linear, SVM Medium Gaussian, SVM Quadratic, Ensemble Subspace KNN, Ensemble Bagged Trees, and Ensemble Subspace Discriminant, all implemented with MATLAB Classification Learner App. AUC and *p* values were calculated as described above. Training and fivefold cross-validation were repeated 100 times. Average AUC and *p* values are reported in Additional file 2.

### Prioritization of genes with the highest predictive value for classification

To rank genes based on their predictive value, randomized elastic net was performed. Randomized elastic net was implemented as a modification of randomized lasso [48] where the lasso technique was replaced with elastic net as follows: for selected *λ*, and *α* = 0.5, data were permuted by adding random penalty factors from the interval [1/*α*,1] for each predictor; model coefficients were estimated (elastic net); 10,000 permutations were performed; and predictors with non-zero coefficients in at least one of the 10,000 permutations were selected (Additional file 2).

### Measurement of the proteins in CD and UC

We measured the levels of CXCL1, CXCL8, CXCL11, CCL20, TNF-α, and IL-1β (see above) in the serum of 15 UC patients, 11 CD patients, and 20 healthy controls using the U-PLEX Biomarker Group 1 (hu) assays, SECTOR (1PL) (Meso Scale Discovery, Rockville, MD, USA; cat. no. K15067 L-1) according to the manufacturer’s instructions. The U-PLEX technology (https://www.mesoscale.com/en/products_and_services/assay_kits/u-plex_gateway) can measure a maximum of 10 proteins at the same time and requires only 25 μl from each sample. The biomarker group used (cat. No. K15067 L-1) was custom designed and only measured the six proteins of interest stated above.

### Drug target analysis of the shared interaction neighborhood

In order to test the therapeutic relevance of the SIN, we downloaded all drugs that had at least one known target in human cells (*n* = 1790; out of which 1408 were approved) from approved and investigational drugs from DrugBank (version 5.0.3). We then tested whether the SIN was enriched for these drugs and found the SIN genes to be significantly enriched for these drug targets (*n* = 302; Fisher exact test OR 2.92; *p* = 2 × 10^−53^). An important therapeutic implication is that drugs targeting the SIN can be potentially used for more than one disease.

### Identification of drugs suitable for targeting the SIN

In order to identify drugs suitable for targeting the SIN, we computationally predicted which of the 1790 drugs would mainly target disease genes in the SIN. The predictions were based on prioritization of drugs in network proximity to the disease genes within SIN [49]. Briefly, we calculated the distance between direct drug targets (*T*) and disease genes (*G*) within the SIN (*M*) on the PPIn [47] using the shortest path distance measure (*d):*

$$ {d}_d\left(G,T\right)=\frac{1}{\left\Vert T\right\Vert }{\sum}_{t\in T}{\min}_{g\in G}d\left(g,t\right). $$

Disease genes were defined as DEGs between patients and controls, as well as genes harboring genetic variants associated with each disease.

To assess the significance of the distance between drugs and disease-associated genes in the SIN (*dd*), we created a reference distribution corresponding to the expected distances using randomly selected groups of drug targets and disease genes. We performed 1000 degree-preserving randomizations using a binning approach that grouped nodes with a certain degree interval together, such that there were at least 300 nodes in a bin. Next, the average *d* and standard deviation of reference *d* distribution were used to convert observed distance to a normalized distance:

$$ z\left(G,T\right)=\frac{d_d\left(G,T\right)-{\mu}_{d_d\left(G,T\right)}}{\sigma_{d_d\left(G,T\right)}}. $$

Subsequently, *z*-scores were transformed to *p* values using MATLAB *ztest()* function, left-sided test (Additional file 2). To further validate these results, we also modified the approach from Guney et al. [49] by calculating the distance between direct drug targets (*T*) and disease genes (*G*), and between disease genes and the SIN (*M*) on the PPIn using the minimum shortest path distance measure *d*

$$ {d}_{dm}\left(G,M,T\right)=\frac{1}{\left\Vert T\right\Vert }{\sum}_{t\in T}{\min}_{g\in G}d\left(g,t\right)\cdotp \left(1+\frac{1}{\left\Vert G\right\Vert }{\sum}_{g\in G}{\min}_{m\in M}d\left(m,g\right)\right). $$

This modification takes into consideration disease genes (defined as described above) that are not a part of the SIN. To assess the significance of the distance between drug, disease genes, and the SIN (*ddm*), we performed 1000 degree-preserving randomizations using binning approach, as described above. Next, the average *d* and SD of reference *d* distribution was used to convert observed distance to a normalized distance:

$$ {z}_m\left(G,M,T\right)=\frac{d_{dm}\left(G,M,T\right)-{\mu}_{d_dm\left(G,M,T\right)}}{\sigma_{d_{dm}\left(G,M,T\right)}} $$

and *z*-scores were then transformed to *p* values as described above (Additional file 2).

We identified five such drugs, which targeted nine out of 13 diseases. We validated one of these drugs, the peroxisome proliferator-activated receptor (PPAR)-alpha agonist bezafibrate, in a T cell-dependent mouse model of rheumatoid arthritis described above, which was not among the 13 diseases that were analyzed to construct the SIN.

### Treatment study of bezafibrate in a mouse model of RA

To test if bezafibrate could dampen inflammation, we administered bezafibrate to 8- to 20-week-old female 129/SvE mice subjected to the antigen-induced RA model, as described above. Bezafibrate (Sigma-Aldrich, B7273) was dissolved overnight in dimethyl sulfoxide (DMSO; 0.1 g bezafibrate/mL) and further diluted in PBS before treatment. Three different treatment protocols were used to test the effect of bezafibrate on arthritis development: (1) systemically at pre-sensitization days 1 and 7 (8 mg bezafibrate/kg included in the immunization solution containing mBSA + Freund’s adjuvant, *n* = 5), (2) systemically by injections after triggering of arthritis (4 mg bezafibrate/kg in a total volume of 500 μL PBS, intraperitoneally on days 21, 24, and 26, *n* = 4), and (3) locally by a single intra-articular injection (0.6 mg bezafibrate/kg included in the mBSA solution used to trigger arthritis, *n* = 5). For all treatments, control mice subjected to antigen-induced arthritis (AIA, *n* = 5) were injected in the same manner with the same volume of PBS/DMSO used for bezafibrate delivery (max 0.1% DMSO). Systemic delivery of bezafibrate after triggering of arthritis prevented the development of arthritis (Additional file 1: Figure S10). Next, we examined if bezafibrate delivery would have local effects. This was done by local treatment with a single intra-articular injection of bezafibrate. We also examined the effects of bezafibrate delivery at antigen sensitization: neither treatment locally or at sensitization had any ameliorating effects on arthritis (Additional file 1: Figure S10). To validate the effects of bezafibrate on T cell proliferation, we proceeded with a proliferation assay, as described below.

### CD4+ T cell proliferation assay

Spleen and lymph nodes draining sites of injection (axillary and popliteal) were isolated, and single-cell suspensions (splenocytes and lymph nodes combined) were prepared by passing the spleen and lymph nodes gently through a 70-μm cell strainer. Red blood cells were lysed by adding RBC lysing solution (Sigma-Aldrich) according to the manufacturer’s instructions. Carboxyfluorescein diacetate succinimidyl ester (CFDA-SE, Sigma-Aldrich) at 5 μM was added to cells and incubated for 5 min in the dark at room temperature (RT, 25 °C). This was followed by washing the stained cells five times with FACS buffer (PBS + 1% FBS). Stained cells (2 × 10^6^/mL in a total volume of 200 μL) from mice subjected to AIA, mice subjected to AIA, and treated with Bezafibrate as described above and from naïve control mice were stimulated with mBSA (50 μg/mL) and cultured for 72 h at 37 °C in 5% CO_2_ and 95% humidity. After 72 h, cells were harvested and analyzed for diminished carboxyfluorescein succinimidyl ester (CFSE) stain by FACS. CFSE-stained, non-stimulated cells from a naïve mouse were used to define non-proliferating cells [50].

### Proliferation assay

To determine the effect of bezafibrate on T cell proliferation in the RA model, spleen, axillary, and popliteal lymph node cells from naïve, bezafibrate-treated, and non-treated controls subjected to AIA were stained with 5 μM CFDA-SE (Sigma-Aldrich) by incubation for 5 min in the dark at RT. Stained cells were washed five times with FACS buffer (PBS + 1% FBS). Stained cells (2 × 10^6^/mL in a total volume of 200 μL) were stimulated with mBSA (50 μg/mL) and cultured at 37 °C in 5% CO_2_ and 95% humidity. After 72 h, cells were harvested and analyzed for diminished CFSE-stain by FACS; see gating strategy (Additional file 1: Figure S11) CFSE-stained cells from a naïve mouse were used to define non-proliferating cells [50]. Assessment of antigen recall responses of CD4+ T helper cells among spleen and lymph node cells showed that the systemic intraperitoneal treatment with bezafibrate that protected from arthritis also inhibited proliferation of CD4+ T helper cells, which was not the case for bezafibrate treatment locally or at sensitization (Additional file 1: Figure S10). Thus, the protective effect of bezafibrate on arthritis development is contingent on its ability to inhibit T helper cell proliferation.

## Results

### scRNA-seq study of a mouse model of arthritis shows wide dispersion of pathogenic mechanisms in multiple cell types

**Fig. 1:**
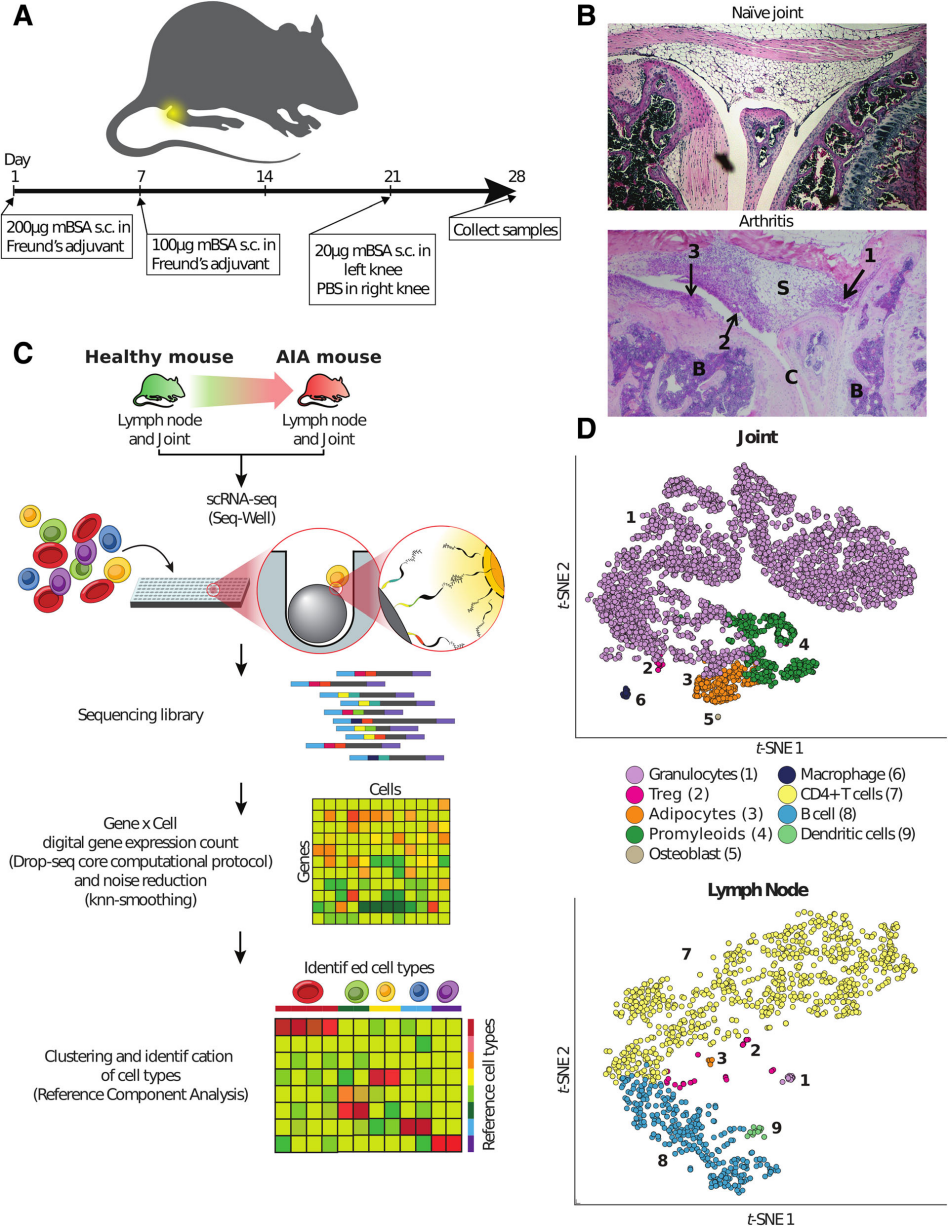
scRNA-seq analysis of a mouse model of antigen-induced arthritis (AIA). **a** An overview of the AIA mouse model. **b** Representative joint images from naïve mice and arthritic joints after hematoxylin and eosin (H&E) staining. B, bone marrow; S, synovial cavity; C, cartilage. Arrows indicate (1) infiltration of inflammatory cells to the synovium, (2) cartilage/bone destruction, and (3) hyperplasia of the synovial lining. **c** A schematic overview of seq-well scRNA-seq and cell type identification using reference component analysis (RCA). **d** t-SNE plot of 7086 healthy and RA joint cells (*n* = 4 healthy mice samples and 5 sick mice samples), and 1333 healthy and AIA lymph nodes cells (*n* = 4 healthy mice samples and 5 sick mice samples), colored by RCA clusters

We performed scRNA-seq analyses of a mouse model of RA, antigen-induced arthritis (AIA). In this model, arthritis is triggered by intra-articular injection of the antigen mBSA in mice previously sensitized with mBSA (Fig. 1a). Histologically, the arthritic tissue resembled that found in human RA, with infiltration of inflammatory cells into the synovium, cartilage/bone destruction, and hyperplasia of the synovial lining (Fig. 1b). scRNA-seq was performed on whole arthritic joints, as well as on joint lymph nodes (Fig. 1c). In total, we recovered 8420 single cells after filtering on a minimum of 10,000 reads and 400 transcripts per cell. Cell type classification was performed using Reference Component Analysis, RCA (see the “Methods” section) [18]. This method uses bulk expression profiles of known cell types as references to classify single-cell profiles based on genome-wide transcriptional similarity. We first tested RCA by analyzing if it correctly classified in-house scRNA-seq data from two cancer cell lines using bulk profiling data from 26 cell lines as a reference and found that this was the case (see the “Methods” section, Additional file 1: Figure S1). We next used RCA to classify cell types in our mouse scRNA-seq data. We identified nine cell types in arthritic mice and seven in healthy controls (Fig. 1d, Additional file 1: Figures S12 and S13). The cell types were dendritic cells, CD4+T lymphocytes, T regulatory cells (Treg), B lymphocytes, macrophages, granulocytes, common myeloid progenitor cells (promyeloids), adipocytes, and osteoblasts. These cell types are similar to those primarily or secondarily involved in human RA [23, 51, 52] and are partially similar to cell types identified by scRNA-seq analysis of synovium from patients with rheumatoid arthritis (RA) [23, 52]. In order to identify and prioritize mechanisms for therapeutic targeting, we first identified differentially expressed genes (DEGs) using Monocle. Similar to the scRNA-seq study of human RA [23], DEGs were calculated for each cell type compared to all other cell types in each tissue separately (see the “Methods” section). We performed pathway analysis of the DEGs in AIA using the Ingenuity Pathway Analysis (IPA). The most significant pathways enriched among differentially expressed genes were found in T cells and related to T cell activation and differentiation, e.g., Cd28 signaling in T helper cells (*p* = 2.51 × 10^−12^ and Th1 differentiation (*p* = 5.75 × 10^−9^). These pathways included genes with key roles for activation and differentiation, such as *Il2*, *Itk*, and *Ifngr1*.

Intuitively, therapeutic targeting of genes in the most significant pathways would appear ideal. Indeed, drugs that inhibit Th1- or Th17-like responses have been developed to treat RA [53]. However, those drugs have shown variable efficacy [54]. One reason could be the involvement of multiple other pathways in T cells and other cell types, which are not targeted. Indeed, the other most significant T cell pathways were highly diverse, e.g., calcium-induced T lymphocyte apoptosis (*p* = 1.48 × 10^−10^), Nfat activation (*p* = 2.13 × 10^−9^), Cdc42 signaling (*p* = 5.37 × 10^−8^), Nur77 signaling in T lymphocytes (*p* = 3.47 × 10^−7^), and production of nitric oxide and reactive oxygen species (*p* = 3.55 × 10^−6^). A similar, and non-overlapping, diversity was found in granulocytes, e.g., virus entry via phagocytosis (*p* = 1.29 × 10^−9^), Mtor signaling (*p* = 1.29 × 10^−9^), integrin signaling (*p* = 6.91 × 10^−8^), leukocyte extravasation (*p* = 5.12 × 10^−6^), caveolar-mediated endocytosis (*p* = 1.12 × 10^−5^), and Vegf signaling (*p* = 3.02 × 10^−5^). Further analyses of all differentially expressed genes in all the cell types showed a great variety of pathways, therapeutic targets, and biomarkers: 285 pathways, 263 drugs, and 873 biomarkers were significantly associated with any of the cell types (*p* < 0.05). The median number of pathways per cell type was 46 (0–205), and the median number of cell types associated with each pathway was 2 (1–8), (Additional file 2). This diversity suggested that specific therapeutic targeting of the most significant pathway in one cell type would not suffice because of multiple other pathways in the same or other cell types. Instead, an impractical number of drugs targeting multiple pathways might be needed. Indeed, the number of drugs predicted to target significant pathways in each cell type was 55.5 (0–144), and the number of cell types predicted to be targeted by each drug was 1 (1–8). Only one drug, sirolimus, targeted most of the identified cell types. This is a potent immunosuppressant, which has been tried in refractory cases of RA, but has significant side effects [55]. We repeated the above analyses in cell types identified by scRNA-seq of human synovium from patients with RA [23]. Similar to the AIA mice there was a great diversity of pathways, which were dispersed across multiple cell types. There were also highly significant pathway overlaps between the same cell types in human and mouse data (Additional file 1: Figure S14, Additional file 2). Effective drug targeting of such complex changes is a formidable challenge, which may explain why many patients with autoimmune diseases do not respond to treatment. This highlights the need for novel, systems-level approaches to prioritize cell types and pathways for therapeutic targeting.

**Fig. 2:**
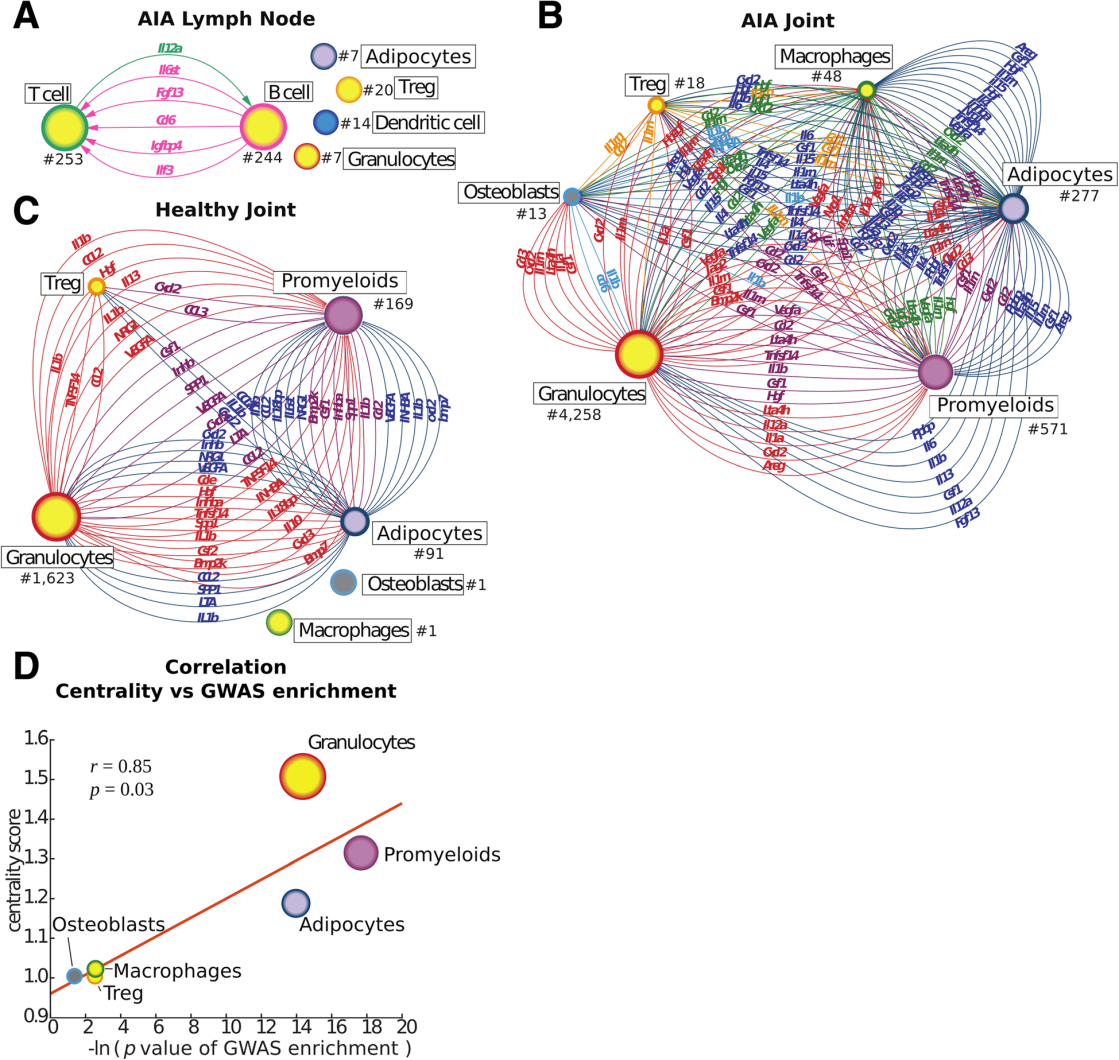
Multicellular disease models (MCDMs) from a mouse model of AIA. MCDMs were constructed based on scRNA-seq data by connecting differentially expressed genes in each cell type with predicted upstream regulators in all other cell types. Cell type size corresponds to centrality score. Numbers indicated by the nodes denote the number of identified cells of specific type (for example in RA joint, we have identified 4258 granulocytes). **a** An MCDM of lymph nodes from arthritic mice. **b** An MCDM from arthritic joints. **c** Multicellular model of a healthy mouse joint (lymph node model is not shown because there was only one predicted interaction). Gene names of predicted upstream regulators are indicated on arrows. Treg, T regulatory cells. **d** Correlation between centrality score of cell types and enrichment of genes harboring genetic variants identified by GWAS and expert curated repositories among differentially expressed genes (the genes were derived from DisGeNet and the analysis based on the mouse orthologues of the human genes)

Here, we examined if network principles could be applied to aid in cell, target, and drug prioritization. Thus, we constructed network models of the cell types in the lymph nodes and joints from arthritic mice—henceforth referred to as multicellular disease models (MCDMs). Interactions were inferred by connecting whole networks of differentially expressed genes in each cell type with their predicted regulators in all other identified cell types. These predictions were based on significantly enriched interactions in the IPA program (see the “Methods” section, Additional file 2) [38]. For example, *Il1b* was predicted as a possible upstream regulator of DEGs in granulocytes. We found that *Il1b* was differentially expressed in promyeloids. This led to the identification of a potential interaction between these two cell types (Fig. 2a–c; Additional file 2). The predicted interactions were supported by similar results using another recently described method (Additional file 2) [40].

In the lymph node, only two cell types, T and B lymphocytes, had predicted interactions (Fig. 2a). By contrast, in the joints, all cell types were connected with each other in a multi-directional manner, mainly by cytokines and chemokines (Fig. 2b). A possible explanation for more interactions in the joints could be a larger number of cells than in lymph nodes (*n* = 7086 and *n* = 1333, respectively). However, we found no significant correlation between the number of cells of each cell type and the number of outgoing edges/interactions (Pearson, sick joint *r* = 0.23, *p =* 0.66; healthy joint *r* = 0.61, *p* = 0.39). Therefore, a more likely explanation is structural differences between lymph nodes and the whole joint. Different cell types in lymph nodes may potentially interact less because they are more localized in dedicated tissue compartments than in joint fluid or tissue. Visual inspection of the resulting MCDM networks revealed no obvious key regulatory cell type, such as a “hub” that had many more interactions than the others, nor an upstream cell type that regulated the others in a linear chain (Fig. 2a, b). Similar results were obtained for MCDMs derived from scRNA-seq from human RA (Additional file 2), as well as when cell types were connected based on another ligand-receptor-based method to infer interactions (see the “Methods” section, Additional file 2) [40]. This suggested that pathogenic mechanisms were dispersed across multiple cell types. In support of multicellular pathogenesis, the differentially expressed genes in most cell types in scRNA-seq data from both mouse arthritis and human RA were significantly enriched for genes harboring genetic variants associated with RA. Those genes were derived from the DisGeNet database [44], and the mouse analyses based on the mouse orthologs of those genes (see the “Methods” section, Additional file 2).

Below, we present data supporting that multicellular pathogenesis is a general characteristic of complex diseases and that network analysis in combination with converging biomedical data can be used to prioritize the most relevant cell types and genes for diagnostics and therapeutics.

### Analyses of MCDM network properties and enrichment of RA-associated genetic variants in mouse arthritis supported that the relatively most important cell types can be prioritized

Because of the complex, multi-directional interactions in the MCDMs, we examined if network centrality could help to prioritize the relatively most important cell types. A detailed explanation of centrality is given in the “Methods” section [39, 42]. Briefly, centrality is a measure of interconnectivity, and our assumption was that the most central cell types would be relatively most important for pathogenesis. We used random walk centrality as a metric for centrality. For robustness, we also included subgraph centrality analysis, and a complementary annotation source to Ingenuity ([43], see the “Methods” section) which for each case showed similar results as the random walk centrality (Additional file 4). As a positive control, we started by analysis of scRNA-seq from human colorectal tumors [18]. We hypothesized that tumor cells would be more central than surrounding stromal and immune cells. To characterize surrounding cell types, we used RCA and generated a reference expression profiling compendium (see the “Methods” section). We constructed an MCDM and found that tumor cells were most central, followed by immune and stromal cells (Additional file 1: Figure S3). We continued by analyses of centrality in the mouse MCDM. The relevance of centrality as an indication of the pathogenic importance of the MCDM cell types was supported by a significant correlation between centrality and the degree of enrichment of genes associated with RA by genetic variants among differentially expressed genes in each cell type (Pearson *r* = 0.85, *p* = 0.03) (Fig. 2d).

### An MCDM of human RA supported multicellular pathogenesis and centrality to prioritize cell types

In order to test the translational value of centrality, we constructed an MCDM based on scRNA-seq data from human synovium from RA patients [23]. We found a significant correlation between centrality and the degree of enrichment of genes harboring genetic variants associated with RA (Pearson *r* = 0.57, *p* = 0.04).

**Fig. 3:**
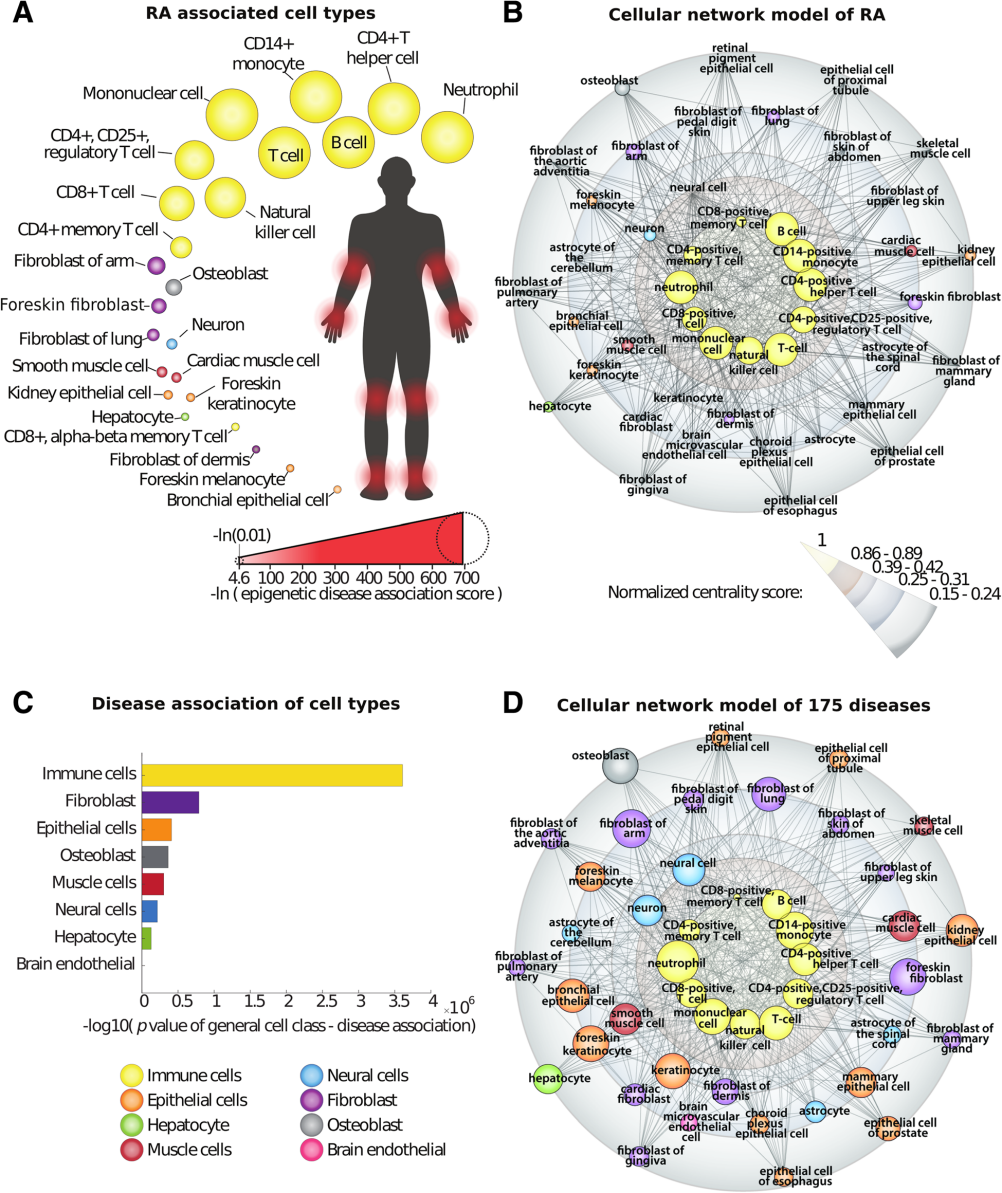
Network models of disease-associated cell types. **a** 24 cell types and subsets that were significantly enriched for GWAS-enriched epigenetic markers associated with RA. Cell type size corresponds to association −ln (*p* value). **b** Network model of cell types associated with human rheumatoid arthritis (RA). Nodes correspond to cell types, node size corresponds to significance of association (−log10 RA GWAS-epigenetic marker enrichment *p* value). Cell types with potential spatial interactions are linked, and cell type position depends on the centrality score as indicated by the rings in the background. **c** Bar plot of cell type classes ordered by significance of association with 175 human diseases (Fisher combined GWAS-enriched epigenetic markers – disease association *p* value calculated for each cell type class). **d** Network model of cell types associated with 175 diseases, based on the same parameters as in **b** (for details see results)

A possible explanation for the lower correlation coefficient in the human synovium MCDM compared to AIA could be that the human MCDM lacked a predominant cell type in RA, namely granulocytes. By contrast, this cell type category was among the most central and also most enriched for genes harboring RA-associated genetic variants in mouse arthritis. This emphasizes the importance of performing scRNA-seq analysis on whole organs to identify pathogenic cell types. However, such analysis may be complicated by not knowing all organs and cell types involved in many diseases. In order to obtain an estimate of cell types and organs involved in human RA, we analyzed cell-type-specific epigenetic markers that were enriched for genetic variants associated with RA (henceforth referred to as GWAS-enriched epigenetic markers; see the “Methods” section; Additional files 2 and 5). The epigenetic markers were identified in the Encyclopedia of DNA Elements [56]. These markers included both activating and repressive elements identified in 45 primary human cell types. This resulted in the identification of 24 cell types and subsets that could be ordered based on their significance of association (Fig. 3a, Additional file 2). The cell types belonged to two main categories, immune cells and local stroma or parenchymal cells.

As expected, immune cell types were most significantly associated. The latter category pointed to organs known, or thought to be involved in RA, namely the joints, lungs, heart, skin, and liver. Some of these are difficult to study in human patients, which led us to ask if centrality could be used to further prioritize cell types and thereby organs. To explore the centrality of these categories, we would ideally need the expression profiles of each cell type to infer molecular interactions and construct a cellular network. Since many of the cell types are not possible, or difficult to obtain from patients, we instead used potential spatial interactions as a simple and binary proxy for molecular interactions. For example, a T cell can spatially interact with both bone and kidney cells, but the latter cannot interact with each other. These predicted interactions were inferred based on manual curation (Additional file 2). Using these interactions, we constructed a cellular network model of human RA (Fig. 3b). In this model, cell type size corresponded to relative pathogenic importance, as defined by significance of GWAS-enriched epigenetic markers, and position in the network to centrality. The model indicated that centrality could potentially be used to prioritize cell types. Similar to the mouse and human scRNA-seq MCDMs, the central cell types were mainly from the immune system, while the peripheral ones were local parenchymal or stromal cell types from different tissues (Fig. 3b). Returning to the question if centrality could help to prioritize between local cell types, osteoblasts were actually less central in the model than, for example, epithelial cells and fibroblasts from the lungs. While this may seem unexpected given the importance of joint involvement in RA, this agrees with immune reactions in the lungs being proposed to have a primary pathogenic role [51]. Taken together, these findings supported the dispersion of pathogenic mechanisms in multiple immune and local tissue cell types in RA.

### A network model of 175 diseases supported multicellular pathogenesis and centrality to prioritize cell types

Our analyses of AIA and RA led us to ask if multicellular pathogenesis is a general characteristic of human diseases and if the most important cell types can be identified in each disease. To address the first question, about multicellular pathogenesis, we identified cell types that were significantly associated with 175 diseases that had been analyzed with GWAS. This was done using GWAS-enriched epigenetic markers, as described above for RA (Additional files 2 and 5). We found that the diseases were associated with a median of 20 (0–45) cell types, which could be potentially ranked in order of relative importance, based on significance of association (Fig. 3c). This ranking showed that immune cells were more significant than local stroma and parenchymal cells, and was supported by pathway analysis of genes harboring genetic variant associated with the 175 diseases, which showed that the Th1 and Th2 activation pathway was most significant (*p* = 3.22 × 10^−34^, Additional file 5), followed by other immune-related or general pathways. Even after removal of immune diseases, the Th1 and Th2 activation pathway remained significant (*p* = 3.3 × 10^−14^; see the “Methods” section). Next, we examined if centrality analysis could be applied to prioritize the most important cell types in human diseases. To formally test if there was an association between cellular centrality and disease risk, we constructed a single, multicellular network model of the 175 diseases analyzed with GWAS (henceforth referred to as GWAS diseases). We used the same construction principles as for the model of RA (Fig. 3d, see the “Methods” section). In support of centrality as an explanation for increased disease risk, we found a significant correlation between centrality and GWAS-enriched epigenetic markers (Pearson *r* = 0.51, *p* = 3.5 × 10^−4^).

In summary, the above analyses supported multicellular pathogenesis as a general disease characteristic, and the potential to prioritize cell types using GWAS-enriched epigenetic markers and centrality. Thus, cellular network models of individual diseases, like the one for RA, may help to prioritize organs or cell types to construct scRNA-seq-based MCDMs from human patients. To facilitate such studies, we have provided network models of each of the 175 diseases, as well as the underlying data (Additional files 2 and 5).

### High cell type interconnectivity implies diagnostic potential of central and clinically accessible cell types

**Fig. 4:**
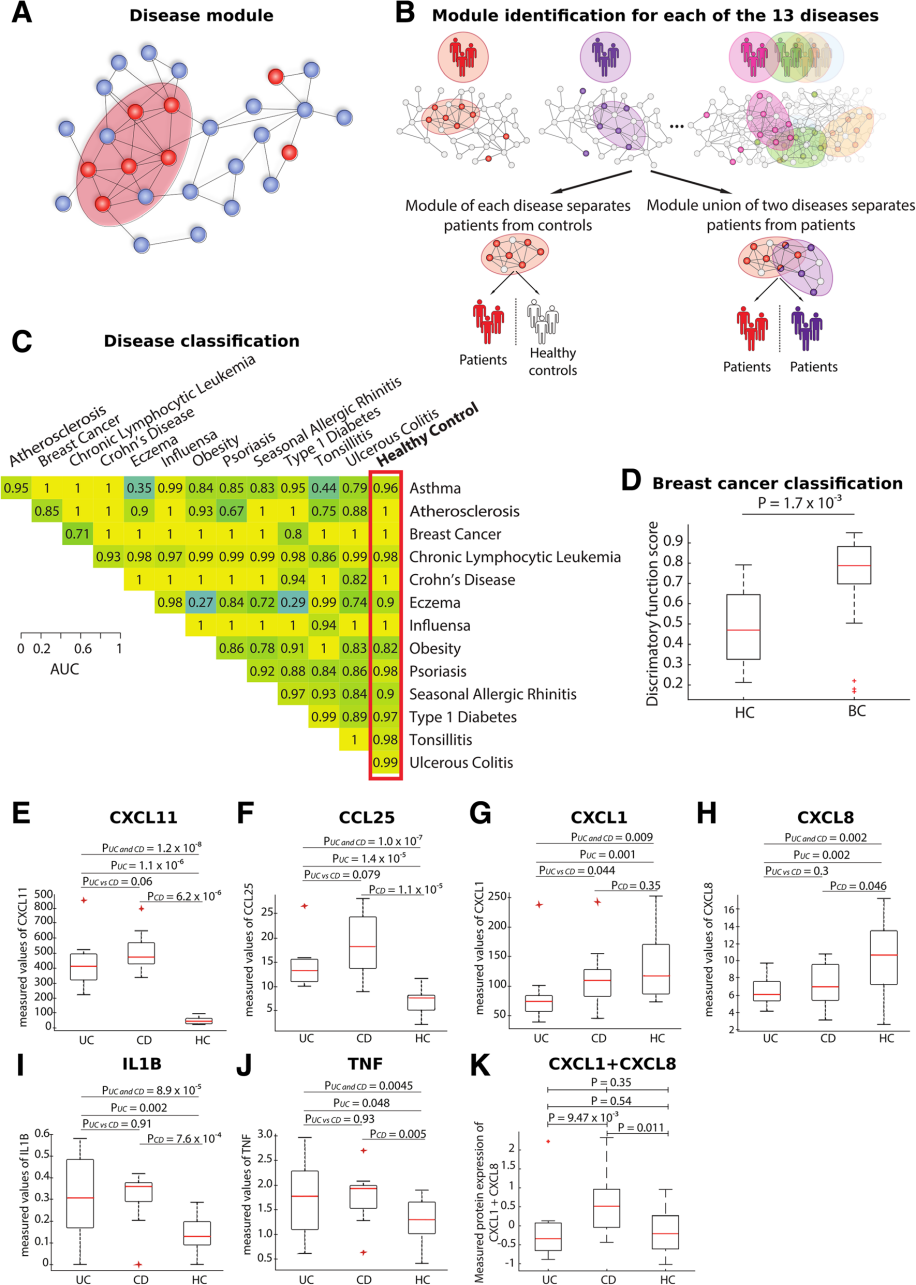
Diagnostic potential of CD4+ T cells based on clinical profiling studies of 13 diseases. **a** Toy model of a disease module. Disease-associated genes (red) are mapped on proteins (blue) in the human protein-protein interaction network. Disease-associated genes that co-localize form a module. **b** Overview of the module-based analyses. First step is the identification of disease modules for each of the 13 diseases profiled in the prospective microarray study of CD4+ T cells. For each disease module, genes separate patients from healthy controls. For pairwise comparison of the diseases, genes in the union of two respective modules separate patients with different diseases; for example, genes in influenza and asthma modules separate patients with influenza from patients suffering from asthma with AUC of 0.99, *p* = 3.3 × 10^−5^, as shown in **c**. **c** Heatmap presenting area under the curve (AUC) values of 13 disease classifications based on the module intersections genes, using elastic net. **d** An independent validation study of classification accuracy of breast cancer patients (*n* = 24) and healthy subjects (*n* = 14) based on previously preselected biomarkers (genes) measured in CD4+ T cells. Classification was performed with elastic net, preserving same lambda (*λ*) value as estimated for the original study. **e**–**j** Potential diagnostic classification of IBD patients based on six secreted plasma proteins identified in the intersection of ulcerative colitis (UC) and Crohn’s disease (CD) modules. These proteins could separate patients from healthy controls (HCs). **e** CXCL11; **f** CCL25; **g** CXCL1; **h** CXCL8; **i** IL1B; **j** TNF. **k** Crohn’s disease and ulcerative colitis patients’ classification based on normalized protein levels of CXCL1 and CXCL8. UC, ulcerative colitis; CD, Crohn disease; HC, healthy controls. Star denotes *p* value < 0.05. **d**–**k** The bars in the boxes represent median and 25th and 75th percentiles, while whiskers extend to ± 2.7*σ* (see the “Methods” section)

Both the MCDM of RA and the network model of 175 diseases supported multicellular pathogenesis. While this complicates therapeutic targeting, the centrality analyses indicated a potential diagnostic advantage: because of its interconnectivity with other cell types, any single, central cell type could serve as a diagnostic sensor of all other disease-associated cell types in an MCDM. We examined this possibility in public microarray data from some central cell types in RA patients, namely CD4+ T cells, B cells, and CD14+ cells. To prioritize between the large number of differentially expressed genes between RA and controls in each cell type, we identified so called modules, i.e., genes that co-localized on the protein-protein interaction (PPI) network (Fig. 4a) [2]. We found that module genes separated patients and controls with high accuracy (area under the curve, AUC_CD4+ T cells_ = 1.0, *p* = 5.4 × 10^−4^, module size = 43; AUC_CD14+_ = 0.74, *p* = 3.4 × 10^−3^, module size = 8; AUC_B cells_ = 0.88, *p* = 4.9 × 10^−3^, module size = 35).

This led us to examine if a central, functionally relevant and clinically accessible cell type could be used diagnostically in clinical studies of human patients with multiple diseases. We focused on expression profiling of the CD4+ T cell because of its centrality, accessibility in peripheral blood, and the pathway analyses of the 175 diseases, described above.

### Prospective clinical studies of 13 diseases demonstrate diagnostic potential of CD4+ T cells

Collection of T cell expression profiling data across multiple diseases is a considerable challenge since it requires involving specialists from primary, secondary, and tertiary care, laboratory facilities to define inclusion/exclusion criteria, as well as a standardized protocol for T cell isolation and analysis. To our knowledge, such a study has not been previously undertaken. Here, we performed such a study of 13 different diseases to evaluate the diagnostic potential of peripheral, total CD4+ T cells, using a highly standardized protocol. The results were validated by independent studies. We analyzed autoimmune, allergic, infectious, malignant, endocrine, metabolic, and cardiovascular diseases (see the “Methods” section), and age/gender-matched controls.

The diseases were Crohn’s disease, ulcerative colitis, psoriasis, seasonal allergic rhinitis, asthma, atopic eczema, acute tonsillitis, influenza, breast cancer, chronic lymphocytic leukemia, type 1 diabetes, obesity, and atherosclerosis. In order to prioritize between differentially expressed genes in each disease, we constructed modules (Fig. 4b). For all but one of the 13 diseases, expression of module genes separated patients from controls with high accuracy (Fig. 4c, marked with red box, median of area under the curve, AUC, was 0.98, range 0.82–1; median *p* < 2.8 × 10^−5^, range 8.5 × 10^−8^ to 7.8 × 10^−4^; Additional file 2). One disease was separated with less high accuracy, namely obesity (AUC = 0.82). While obesity is increasingly recognized as an inflammatory disease [57], a likely explanation is that it has a greater metabolic component than the other investigated diseases.

The classifications were not dependent on the classification method since similar results were obtained using 20 different methods (Additional file 2). As an example, the AUC for breast cancer was 1, *p* = 10^−4^. An independent validation study in 24 breast cancer patients and 14 healthy subjects yielded an AUC of 0.82; *p* = 1.7 × 10^−3^ (Fig. 4d), which was significantly higher compared to random genes (*p* < 3.7 × 10^−258^).

We also found that the disease module genes separated patients with different diagnoses from each other (Fig. 4c, median AUC 0.98, range 0.27–1; median *p* < 1.0 × 10^−3^, range 1.32 × 10^−5^ to 0.69; Additional file 2). As an example, the inflammatory bowel diseases ulcerative colitis (UC) and Crohn’s disease (CD), which may be difficult to separate in clinical practice [58], were classified with a cross-validated AUC of 0.82 (*p* < 0.03)*.* Taken together, our analyses of 13 different diseases support the diagnostic potential of CD4+ T cells. However, since expression profiling of T cells is complex in clinical settings, we searched for module intersection genes that encoded secreted proteins, which could more readily be measured diagnostically in sera. We identified six such proteins in UC, CD, and healthy controls (*n* = 15, 11, and 20, respectively). All six proteins were differentially expressed in patients versus controls (non-parametric Wilcoxon test had 10^−8^ < *p* < 4.5 × 10^−3^, Fig. 4e–j). Using random elastic net, we ranked those module intersection genes by their predictive value in discriminating patients with CD from patients with UC. We proceeded and aimed for a combination of biomarkers to separate UC and CD. For this purpose, we applied our previously described strategy [59] that avoids any additional parameter inferences. Therefore, we expect the results to be reproducible for a new validation cohort. We normalized each protein to have unit variance and zero mean in the healthy controls and tested whether the sum of the normalized levels separated UC and CD. We found that summing the two proteins with highest individual predictive values (CXCL1 and CXCL8, Additional file 2) separated UC and CD with an AUC of 0.81 (double-sided *p* = 9.47 × 10^−3^, Fig. 4k). We emphasize that this simple approach is likely translatable to new studies, as long as control samples exist. For all disease pairs of all 13 diseases, we therefore provide respective gene lists rank-ordered by their predictive value to find highly predictive combinations of protein biomarkers, similarly to what we described for UC and CD (Additional file 2).

### Pleiotropic mechanisms in T cells are highly enriched for genetic variants and drug targets

Next, we analyzed the therapeutic potential of T cells. As described above, this was complicated by the involvement of multiple pathways and drug targets in T cells. Therefore, we needed a complementary principle to prioritize disease-associated genes in this cell type. We hypothesized that, since T cells were associated with multiple diseases, there could be overlapping, pleiotropic, disease mechanisms. If so, those mechanisms could have a relatively greater pathogenic importance and therefore be prioritized for therapeutic targeting. If this could be shown, a general implication could be improved drug prioritization based on analysis of pleiotropic mechanisms in central cell types. Indeed, modules from the 13 diseases partially overlapped on the PPI network, and their union formed what henceforth will be referred to as a shared interaction neighborhood (SIN; see the “Methods” section). The pathogenic and therapeutic importance of the SIN was supported by highly significant enrichment of genes harboring genetic variants associated with the GWAS diseases (*n* = 261 genes, odds ratio (OR) = 2.83, *p* = 1.5 × 10^−37^), as well as drug targets (*n* = 302, OR = 2.92, *p* = 2 × 10^−53^).

### Identification and validation of drugs targeting the SIN

Because the above analyses supported a general pathogenic and therapeutic importance of the SIN, we hypothesized that it could be exploited to identify drugs for that could be effective in many diseases, in which CD4+ T cells had a central role. We computationally tested this hypothesis in the 13 diseases analyzed above, as well as by a therapeutic study of AIA. To find drugs that optimally targeted SIN genes, we used a recently described network-based method [49]. Briefly, we computationally predicted which of the 1790 drugs in DrugBank would mainly target genes that were in network proximity with the SIN genes. We identified five such drugs, which targeted nine of the 13 diseases (Additional file 2). We tested one of these drugs in the mouse model of AIA. The drug, bezafibrate, is a PPARa agonist used to treat hyperlipidemia and to prevent cardiovascular disease [60]. To our knowledge, bezafibrate treatment has not been described in RA. However, positive effects of PPARg agonists have been described [61, 62].

**Fig. 5:**
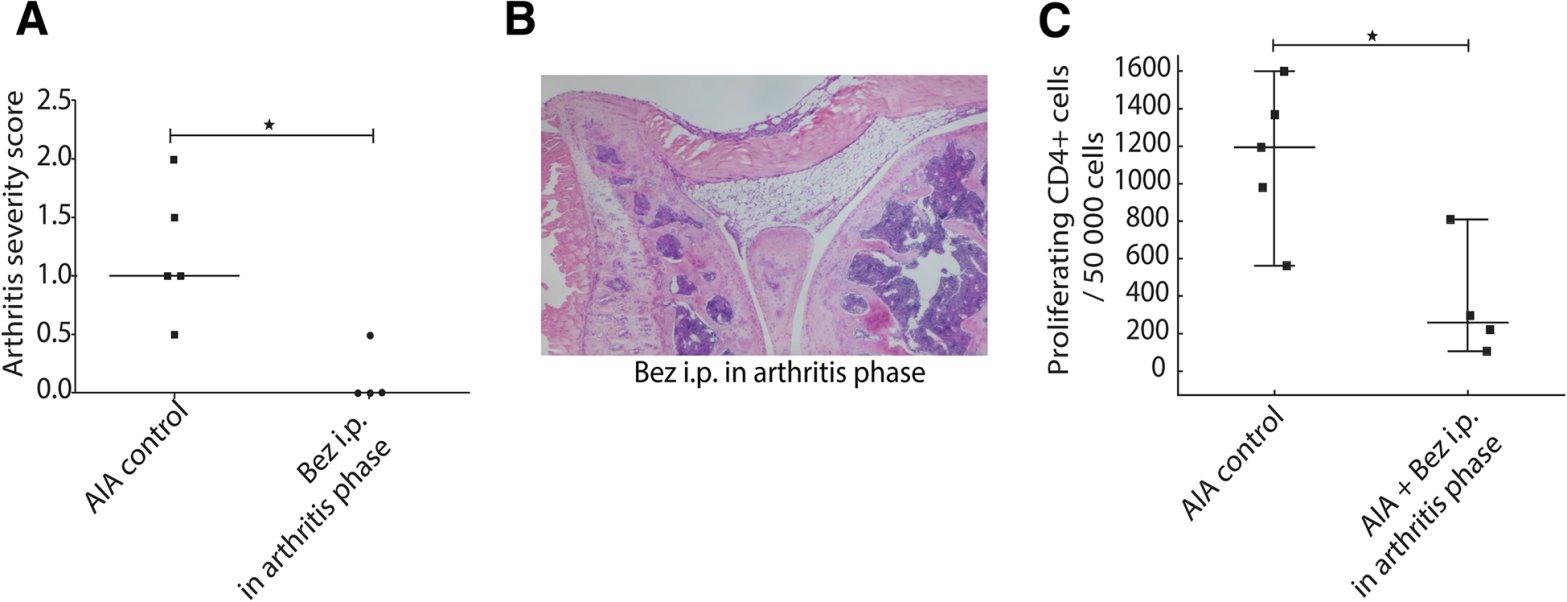
Bezafibrate protects against antigen-induced arthritis (AIA). Female mice with mBSA-induced arthritis were intraperitoneally (i.p.) treated with bezafibrate (*n* = 4) or mock (AIA control, *n* = 5). **a** Arthritis severity was scored based on histopathology day 28 in the two groups (H&E staining, vertical bars indicate median, differences between groups evaluated using the Mann-Whitney *U* test, *p < 0.05). **b** Representative H&E joint image from the bezafibrate-treated mice. **c** Antigen recall response of CD4+ helper T cells among spleen and lymph node cells isolated from mock- (AIA control, *n* = 5) or bezafibrate-treated (*n* = 4) mice; vertical bars indicate mean ± SEM, differences between groups evaluated using the two-sided Mann-Whitney *U* test *p < 0.05)

In our study of the AIA mice, histological specimens from arthritic joints that had or had not been treated with bezafibrate were stained with hematoxylin and eosin and examined in a blinded manner for an arthritis score [33]. This ranged from 0–3, where 0 = no signs of inflammation, 1 = mild inflammation, with proliferation of the synovial lining layer, and 2 and 3 = different degrees of influx of inflammatory cells, as described [33]. We found that bezafibrate treatment significantly decreased the arthritis score (*p* < 0.05; arthritis score in mock-treated control mice median of 1, range 0.5–2.0; bezafibrate-treated mice median of 0, range 0.0–0.5; Fig. 5a, b). Since we have previously shown influx of neutrophils and lymphocytes in untreated arthritis [63], and no inflammatory cells were found in the joints following bezafibrate treatment, our study is the first to show this effect in a mouse model of arthritis. Because the lymphocyte response was induced by a specific antigen, bovine serum albumin (BSA), we could test if this response was affected by bezafibrate. Indeed, we found significantly decreased antigen recall responses in CD4+ T cells in a proliferation assay following bezafibrate treatment (*p =* 0.032; number of proliferating cells in mock-treated control mice median of 1195, range 562–1599; bezafibrate-treated mice median of 259.5, range 107–809; Fig. 5c).

## Discussion

Understanding of pathogenic mechanisms and identification of drug targets in complex diseases are daunting challenges, because of the involvement of thousands of genes in many different cell types. Ideally, it would be possible to identify and target a single key regulatory cell type and mechanism in each disease. Traditionally, such targets are sought based on empirical or screening approaches. However, unbiased genome-wide approaches like GWAS and scRNA-seq studies have indicated the dispersion of multiple pathogenic mechanisms across many cell types [13, 64, 65].

This may explain the difficulties in drug discovery and why many patients do not respond to treatment. Despite this, systematic characterization and prioritization of disease-associated cell types and mechanisms for diagnostics and therapeutics remain unresolved challenges. One obvious approach would be to identify and target the most significant pathway. In our scRNA-seq study of AIA, we found that pathways involved in T cell differentiation were most significant. This is consistent with the current understanding of RA pathogenesis and has resulted in drugs targeting such pathways. However, the effects have been variable. A likely explanation was suggested by our systematic analyses of the scRNA-seq data, which revealed a large number of other pathways and therapeutic targets in each cell type, as well as limited overlap between the cell types. This indicates the need for systems-level approaches to organize and prioritize cell types and mechanisms for diagnostic and therapeutic purposes.

Here, our results support that network-based principles can be applied for both. We organized scRNA-seq data from AIA and human RA into MCDMs. Instead of any unique cell type or mechanism having an obvious key regulatory role, most cell types in the MCDMs interacted, forming multidirectional networks, in which multiple cell types potentially contributed to pathogenesis. Although it is possible that one cell type and mechanism had a key role, our analysis of genetic and epigenetic data supported that pathogenic mechanisms were dispersed in multiple cell types.

This led us to examine if multicellular pathogenesis was a general disease characteristic. Indeed, our analyses of GWAS-enriched epigenetic markers in 175 diseases showed that a median of 20 cell types was associated with each disease. However, those analyses were based on only 45 out of an unknown total number of cell types in the human body. Therefore, the number of disease-associated cell types is likely to be much higher. This complexity emphasized the need for strategies to prioritize the most important disease-associated cell types. Our subsequent analyses supported that such prioritization is feasible, based on centrality analyses of our scRNA-seg-based MCDMs and network models of 175 diseases. We have provided individual models of all the 175 diseases (Additional file 5), as well as the underlying data (Additional file 2), to help prioritization of cell types and tissues for scRNA-seq-based MCDM construction. Such prioritization is important because most complex diseases involve multiple organs, some of which may not be known to be affected in the diseases. For example, the network model of human RA included 24 cell types and cell categories, from different organs. One of these was bronchial epithelium, which is not clinically associated with RA, but recently proposed to have causal role in this disease [51].

While dispersion of pathogenic mechanisms in multiple cell types complicates drug discovery, high interconnectivity between cell types may have an unexpected diagnostic advantage: any central cell type can potentially serve as a sensor of all other disease-associated cell types. This was supported by our analyses of three different immune cells in peripheral blood from RA patients. To our knowledge, this potential advantage has not been previously explored. This would be complicated by the need to perform coordinated clinical studies of multiple diseases according to standardized protocols. In this work, we did perform such studies. We first identified T cells as a suitable diagnostic and therapeutic candidate cell type both because of its centrality and its clinical accessibility. The diagnostic potential of T cells was supported by prospective clinical studies of patients with 13 highly diverse diseases. Expression profiles of this cell type could be used to separate the different diseases from healthy controls and each other, with high accuracy. An independent validation study showed the potential of T cell profiling to diagnose breast cancer. We also found that the profiles could be used to infer a limited number of protein biomarkers for two diseases with partially similar phenotypes, UC and CD. We propose that the data and methods can be used to identify diagnostic proteins for any of the 13 diseases, or newly generated data from other cell types that have been prioritized based on scRNA-seq-derived MCDMs. For this purpose, we have included gene lists rank-ordered by the predictive value that can be used to prioritize biomarkers for further studies. Moreover, based on the example of UC and CD, we describe a method to identify possible combinations of biomarkers from those lists, for better classification accuracy.

While high interconnectivity between cell types was diagnostically advantageous, it complicated prioritization between the many cell types, mechanisms, and drugs. Here, we focused on T cells because of the centrality and GWAS analyses. However, this approach was also challenging due to the involvement of multiple pathways and predicted drug targets in T cells. We hypothesized that, since T cells were associated with multiple diseases, this pleiotropy could be a sign of pathogenic importance. If so, the same pleiotropic mechanisms could potentially be exploited for therapeutic targeting of multiple diseases. This was supported by overlap, the SIN, between modules from the 13 diseases. The pathogenic and therapeutic importance of the SIN was shown by highly significant enrichment of genes harboring disease-associated genetic variants, as well as therapeutic targets. Using network tools, we computationally predicted five out of 1790 drugs that optimally targeted the SIN. We validated one, bezafibrate, in the AIA model. An implication of this study is that drug prioritization may be improved by analysis of pleiotropic mechanisms in central cell types. This is a finding of considerable potential importance that, to our knowledge, is novel and merits further studies.

Limitations of the study include that the bioinformatics analyses of gene interactions, pathways, biomarkers, and drug targets were based on a manually curated aggregate of multiple data sources, which may be confounded by, for example, cell type- or tissue-specific variations. However, we repeated the analyses using independent ligand-receptor data with similar results. The therapeutic implications of our mouse study should be interpreted with caution, since it was based on a standardized protocol and inbred mice of the same age and sex. Indeed, the treatment effects of a related drug were less pronounced in human patients with RA [61, 62]. A likely explanation could be the greater complexity and variability of human RA, as indicated by the pathway analyses of scRNA-seq data from human RA synovium. Thus, combinations of drugs targeting multiple pathways may be required. This highlights the need for future studies aiming at detailed characterization of dispersion of pathogenic mechanisms in MCDM cell types, as well as individual variations. A potential clinical implication is that, for severe diseases that require costly or risky medications, scRNA-based MCDMs may provide a framework to tailor treatments for individual patients, similar to how we now take high-resolution imaging for granted [24].

Another important implication is suggested by recent advances in digital medicine, where different computational methods, such as artificial intelligence, have been applied for automated diagnostics of medical images [66]. Given the molecular complexity of common diseases, successful implementation of digital medicine will require integration of high-resolution molecular data with routine data, such as medical images. We and others have developed network methods for integration of heterogeneous large-scale data, which may prove useful in this context [2, 29].

## Conclusions

Our findings support that MCDMs and network principles may have the potential to prioritize cell types and mechanisms for biomarker and drug discovery. All the presented data and methods have been made available to facilitate such studies.

## Additional files

Additional file 1: Contains **Figures S1–S14.** (PDF 2063 kb)

Additional file 2: A spreadsheet file containing all the data cited in the text in several sheets (ordered by appearance). Description: Description of the content in each sheet in the document. (XLSX 6315 kb)

Additional file 3: Contains **Tables S1–S4.** (PDF 79 kb)

Additional file 4: Random walk betweenness centrality of cell types for validation of MCDM in joint tissue. Contains supplementary table describing ligand-receptor based centrality analyses. (PDF 115 kb)

Additional file 5: Models of 174 diseases based on epigenetic marker enrichments in GWAS genes (excluding rheumatoid arthritis presented in the main text). Compressed folder (ZIP), which can be found as DataS2.zip at: https://figshare.com/articles/DataS2_zip/7976552. (DOCX 14 kb)

## Data Availability

The single-cell raw data generated in this study is publicly available on SRA database (https://www.ncbi.nlm.nih.gov/sra) with accession PRJNA504425. The code for module construction is available at https://gitlab.com/Gustafsson-lab/MODifieR. All codes for analyzing the MCDMs are available at https://gitlab.com/Gustafsson-lab/mcdm_project.

## Abbreviations

AIA: Antigen-induced arthritis
AUC: Area under the curve
BMI: Body mass index
BSA: Bovine serum albumin
CD: Crohn’s disease
CFDA-SE: Carboxyfluorescein diacetate succinimidyl ester
CFSE: Carboxyfluorescein succinimidyl ester
CLL: Chronic lymphocytic leukemia
CRC: Colorectal cancer
DEGs: Differentially expressed genes
DMSO: Dimethyl sulfoxide
ENCODE: Encyclopedia of DNA Elements
GWAS: Genome-wide association studies
H&E: Hematoxylin and eosin
HCs: Healthy controls
i.p.: Intraperitoneally
IBDs: Inflammatory bowel diseases
IPA: Ingenuity Pathway Analysis
mBSA: Methylated bovine serum albumin
MCDMs: Multicellular disease models
NHGRI: National Human Genome Research Institute
OS: Dermatologist
PASI: Psoriasis Area and Severity Index
PBS: Phosphate-buffered saline
PCA: Principal component analysis
PFA: Paraformaldehyde
PPAR: Peroxisome proliferator-activated receptor
PPI: Protein-protein interaction
PPIn: Protein-protein interaction network
promyeloids: Myeloid progenitor cells
RA: Rheumatoid arthritis
RAST: Radioallergosorbent tests
RCA: Reference component analysis
RT: Room temperature
scRNA-seq: Single-cell RNA-sequencing study
SIN: Shared interaction neighborhood
SNPs: Single nucleotide polymorphisms
Treg: T regulatory cells
UC: Ulcerative colitis
