# Correction to: A validated single-cell-based strategy to identify diagnostic and therapeutic targets in complex diseases

**DOI:** 10.1186/s13073-020-00732-7

**Published:** 2020-04-28

**Authors:** Danuta R. Gawel, Jordi Serra-Musach, Sandra Lilja, Jesper Aagesen, Alex Arenas, Bengt Asking, Malin Bengnér, Janne Björkander, Sophie Biggs, Jan Ernerudh, Henrik Hjortswang, Jan-Erik Karlsson, Mattias Köpsen, Eun Jung Lee, Antonio Lentini, Xinxiu Li, Mattias Magnusson, David Martínez-Enguita, Andreas Matussek, Colm E. Nestor, Samuel Schäfer, Oliver Seifert, Ceylan Sonmez, Henrik Stjernman, Andreas Tjärnberg, Simon Wu, Karin Åkesson, Alex K. Shalek, Margaretha Stenmarker, Huan Zhang, Mika Gustafsson, Mikael Benson

**Affiliations:** 1 Centre for Personalized Medicine, Linköping University, Linköping, Sweden; 2 Department of Internal Medicine, Region Jönköping County, Jönköping, Sweden; 3 Departament d’Enginyeria Informàtica i Matemàtiques, Universitat Rovira i Virgili, Tarragona, Spain; 4 Department of Surgery, Region Jönköping County, Jönköping, Sweden; 5 Office for Control of Communicable Diseases, Region Jönköping County, Jönköping, Sweden; 6 Division of Rheumatology, Autoimmunity, and Immune Regulation, Department of Clinical and Experimental Medicine, Linköping University, Linköping, Sweden; 7 Department of Clinical Immunology and Transfusion Medicine, Linköping University, Linköping, Sweden; 8 Department of Gastroenterology and Department of Clinical and Experimental Medicine, Linköping University, Linköping, Sweden; 9 Department of Medical and Health Sciences, Linköping University, Linköping, Sweden; 10 Bioinformatics, Department of Physics, Chemistry and Biology, Linköping University, Linköping, Sweden; 11 Department of Otorhinolaryngology, Yonsei University College of Medicine, Seoul, South Korea; 12 Clinical Microbiology, Region Jönköping County, Jönköping, Sweden; 13 Division of Clinical Microbiology, Department of Laboratory Medicine, Karolinska Institute, Karolinska University Hospital Huddinge, Stockholm, Sweden; 14 Karolinska University Laboratory, Karolinska University Hospital, Solna, Sweden; 15 Department of Dermatology and Venereology, Region Jönköping County, Jönköping, Sweden; 16 Department of Clinical and Experimental Medicine, Faculty of Medicine and Health Sciences, Linköping University, Linköping, Sweden; 17 Futurum – Academy for Health and Care, Department of Pediatrics, Region Jönköping County, Jönköping, Sweden; 18 Institute for Medical Engineering and Science, Massachusetts Institute of Technology, Cambridge, MA USA; 19 Department of Chemistry, Massachusetts Institute of Technology, Cambridge, MA USA; 20 Koch Institute for Integrative Cancer Research, Massachusetts Institute of Technology, Cambridge, MA USA; 21 Broad Institute of MIT and Harvard, Cambridge, MA USA; 22 Ragon Institute of MGH, MIT and Harvard, Cambridge, MA USA; 23 Department of Pediatrics, Institution for Clinical Sciences, Göteborg, Sweden

**Correction to: Genome Med 11, 47 (2019)**

**https://doi.org/10.1186/s13073-019-0657-3**

It was highlighted that in the original article [1] there was an omission in the Funding section. This Correction article shows the correct Funding section.
