# Supplementary material for: A validated single-cell-based strategy to identify diagnostic and therapeutic targets in complex diseases
Source: Genome Med. 2019 Jul 30;11:47. doi: 10.1186/s13073-019-0657-3 (PMC6664760; doi:10.1186/s13073-019-0657-3)
Supplement: Supplementary file 1 — Contains Figures S1–S14. (PDF 2063 kb) [file 13073_2019_657_MOESM1_ESM.pdf]

Supplementary Figures

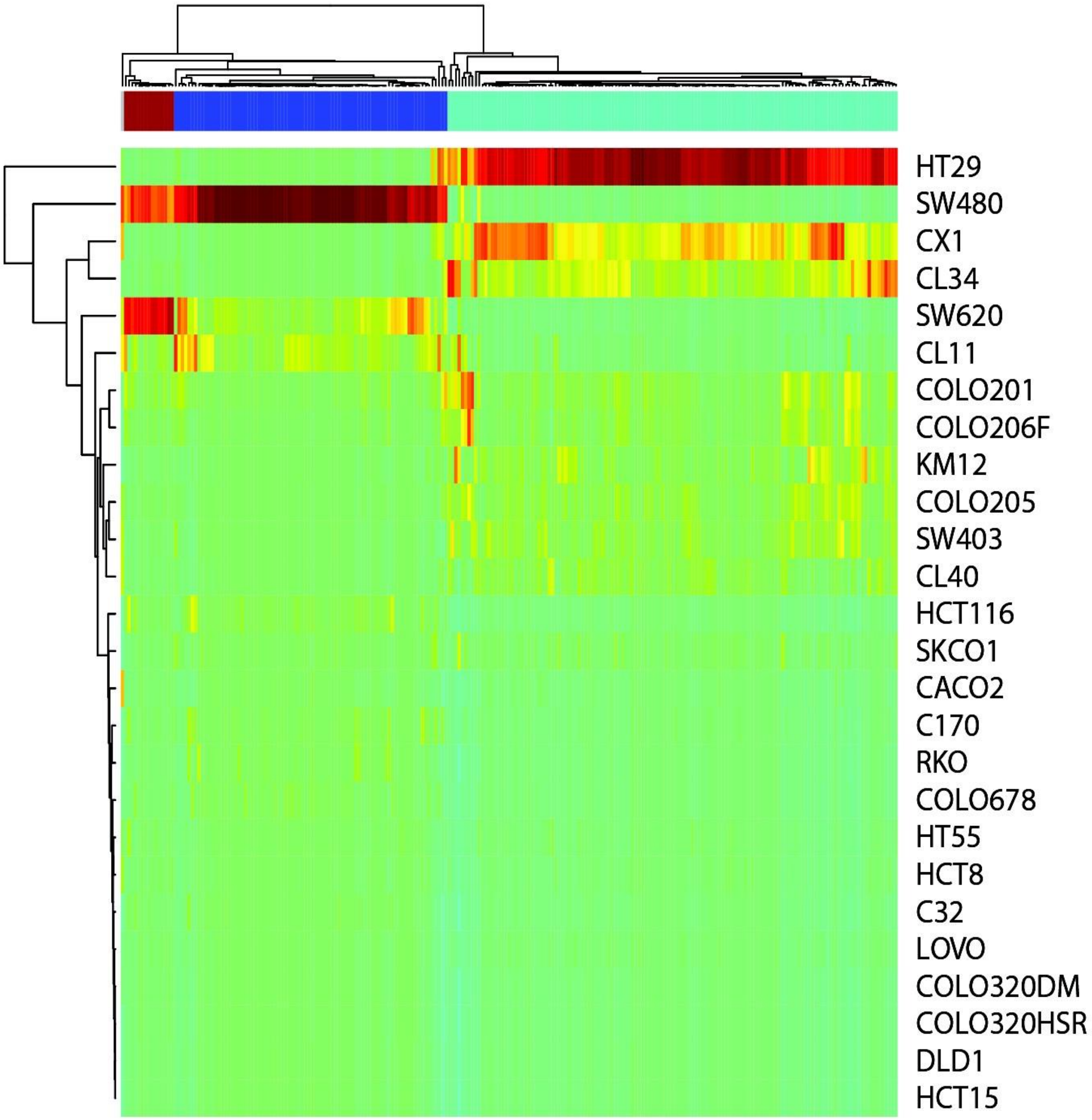

Fig. S1. Reference component analysis (RCA) clustering of in-house single cell data from HT29 and SW480 colorectal cancer cell lines. In order to confirm that our pipeline could distinguish between cell lines, we mixed different colorectal cancer cell lines before loading on the cell array and proceeded according to the previously described single-cell RNA sequencing protocol. We found that we could distinguish between HT29 and SW480, and identify a SW620-like sub-cluster within the SW480 cell population using our pipeline. Both SW480 and SW620 cell lines come from the same patient. We identified three main clusters denoted by the brown, blue, and turquoise colors. The colors of the heatmap, from light blue to dark red, denote the level of similarity towards the reference, where blue means no similarity and dark red means high similarity.

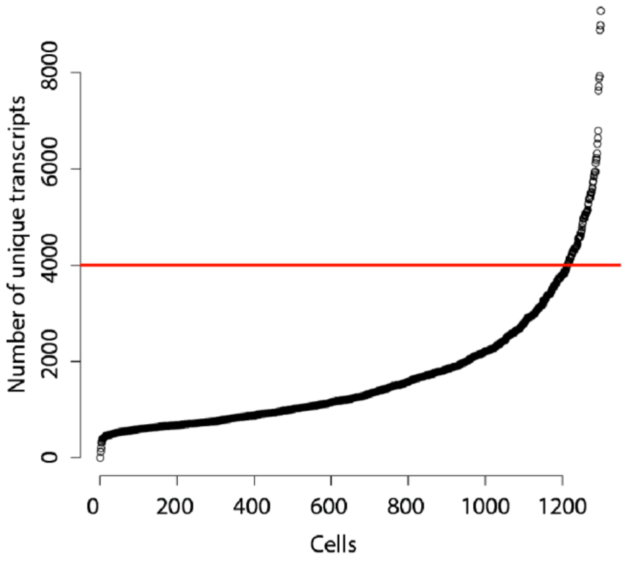

Fig. S2. Gene transcript distribution over individual colorectal cancer cells. Distribution of number of unique transcripts (y-axis) per individual cell (x-axis). Red line shows the selected cutoff point. To select the top quality cells, a threshold around the elbow in which cells increase drastically the number of (unique) transcripts was chosen. To ensure the good quality of the cells as well as the coverage of genes, those cells more than 4,000 unique transcripts were selected for downstream analysis.

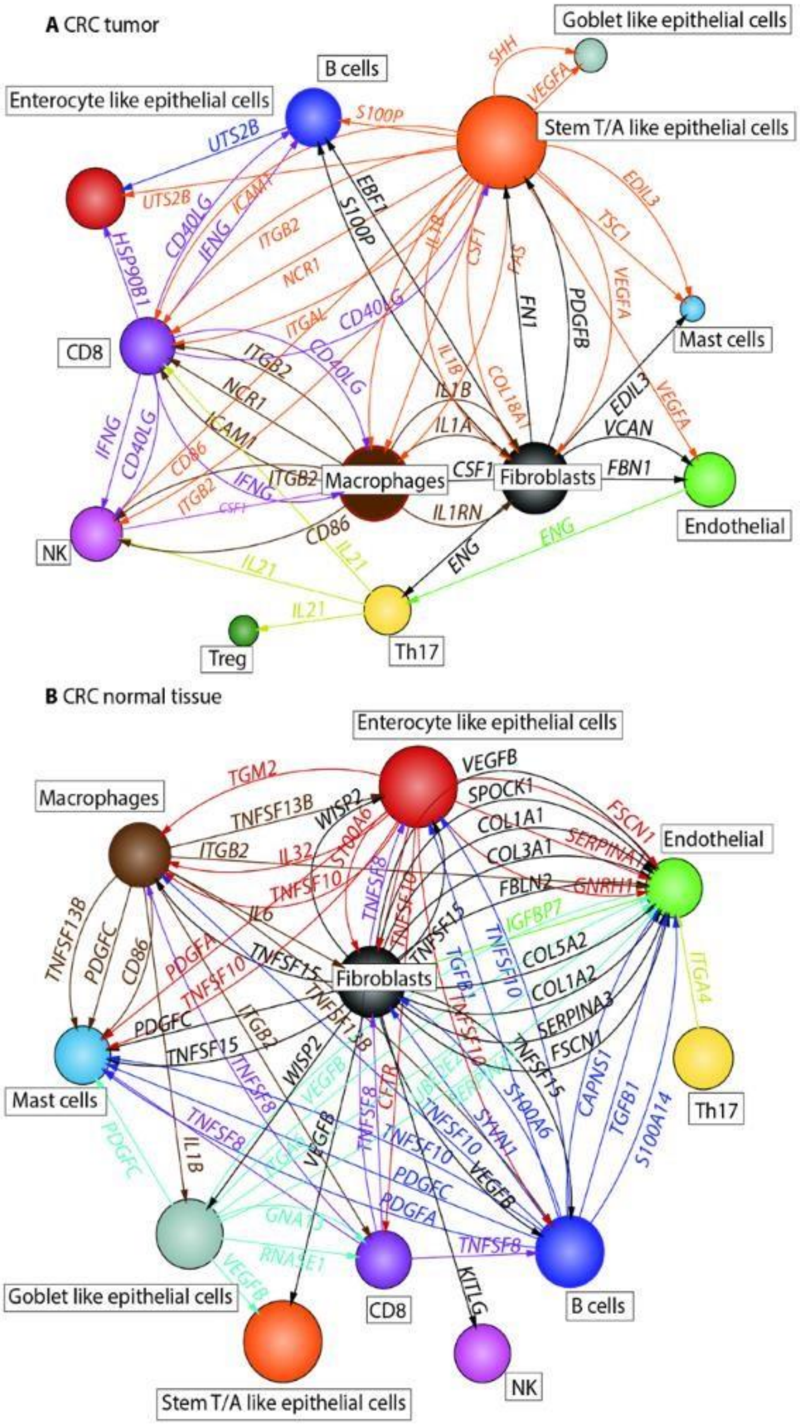

Fig. S3. Multicellular Disease Models (MCDMs) from tumor- and adjacent healthy tissues from patients with colorectal cancer (CRC). (**A**) An MCDM for CRC tumor was constructed, based on connecting differentially expressed genes in each cell type with predicted upstream regulators in all other cell types. Gene names of predicted upstream regulators are indicated on arrows. Cell size corresponds to the number of differentially expressed genes in logarithmic scale. NK = Natural Killer cells, Treg = T regulatory cells. (**B**) Multicellular model from adjacent healthy tissue from CRC patients.

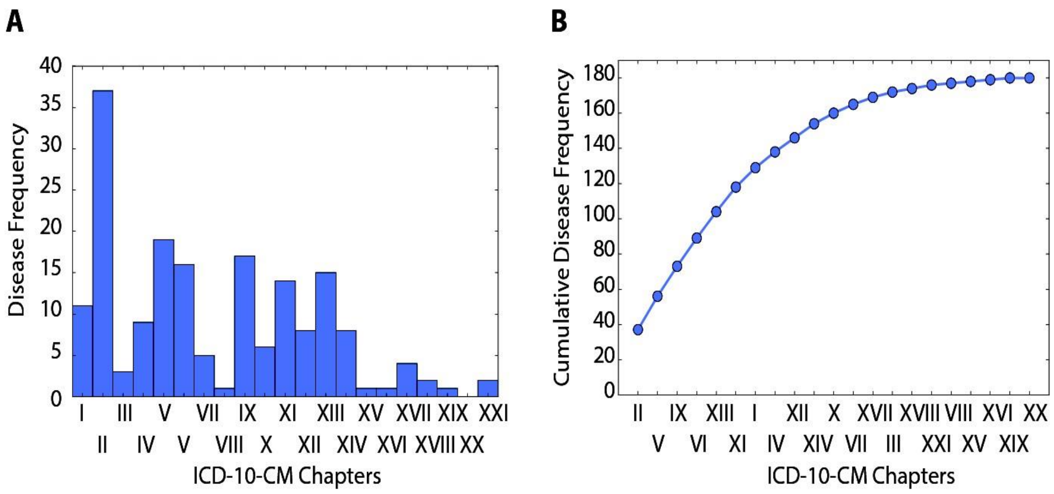

Fig. S4. GWAS diseases distribution over ICD-10-CM chapters. (A) Frequency distribution of the analyzed GWAS diseases across 21 ICD-10-CM chapters. **(B)** Cumulative frequency of diseases across chapters.

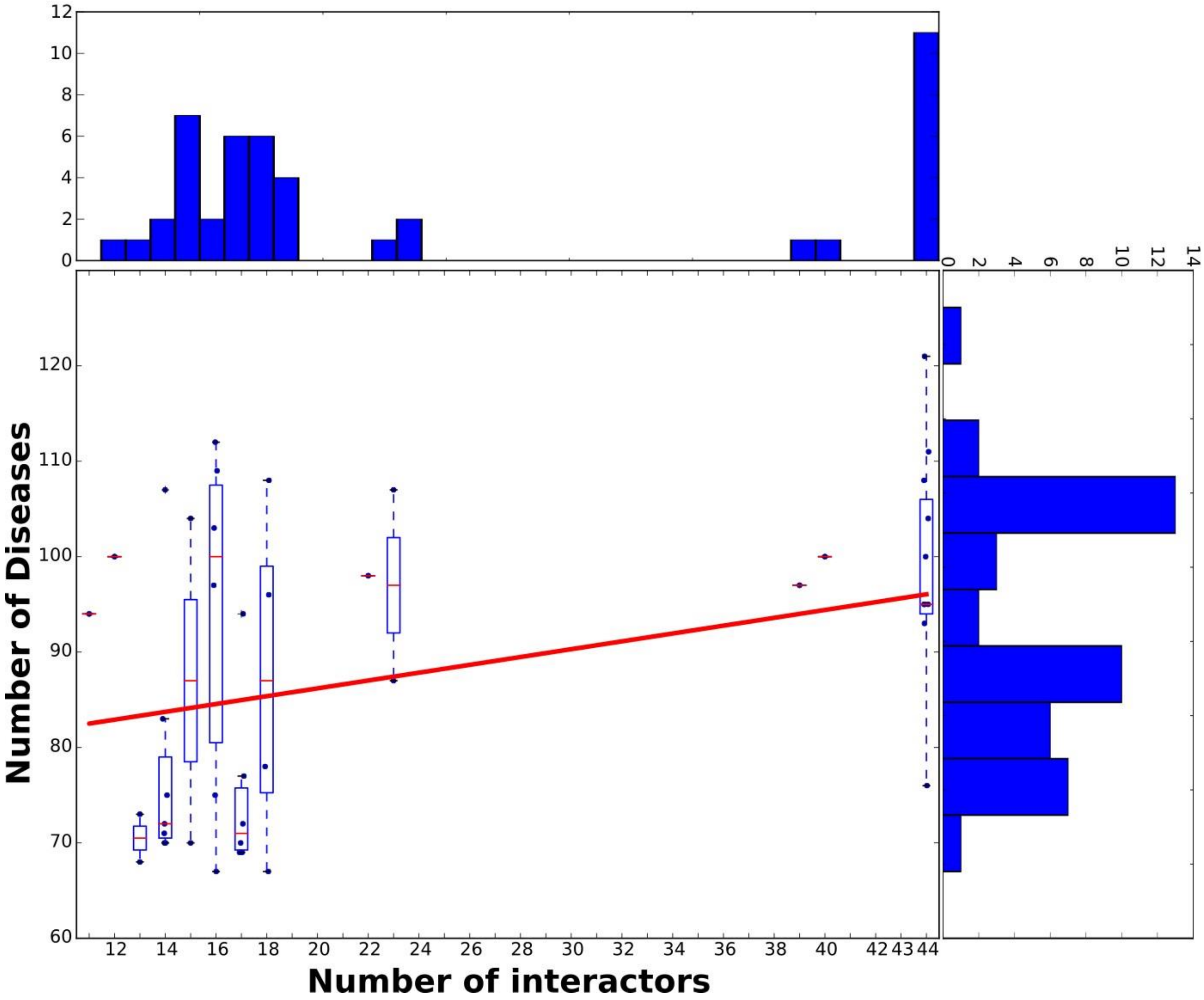

Fig. S5. Relationship between number of interactors of each cell type and the number of associated diseases (CellComp). Correlation between the numbers of interactors and number of associated genes among the cells (Pearson ρ = 0.31, p = 0.038). The top bars show the distribution of the degree distribution (number of interactions for each cell). The side bars show the distribution of number of diseases associated with the cells.

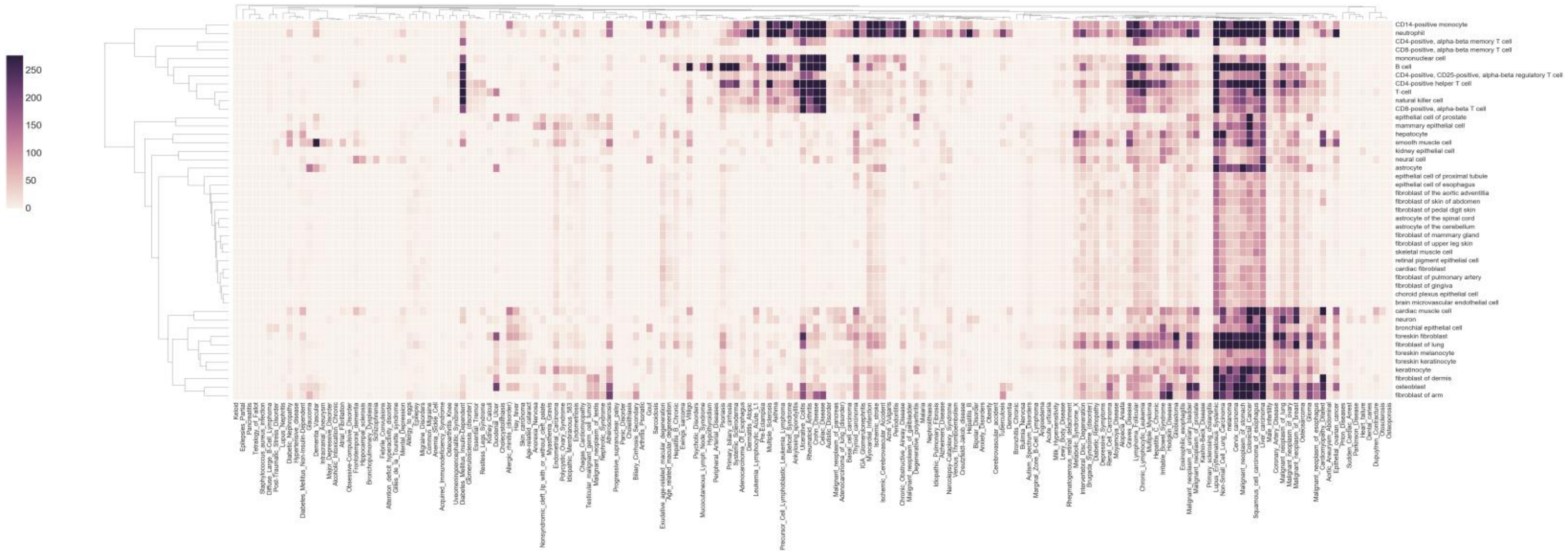

Fig. S6. Cluster diagram showing the associations of 175 diseases with 45 cell types. Relationship between cell type and disease based on epigenetic markers and GWAS data for each cell type and disease respectively. The heatmap shows -log10 of the *p* value for each disease — cell type pair. Hierarchical clustering was performed on these values using the cosine distance over each row pair and column pair.

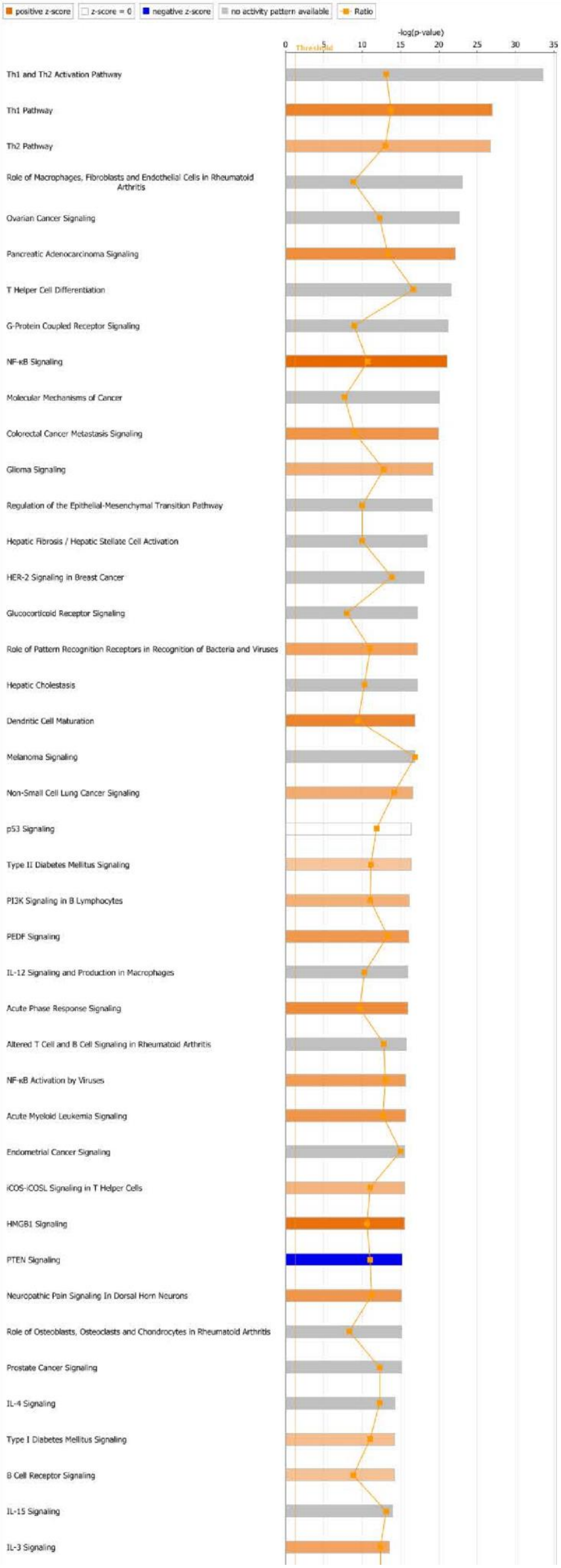

positive z-score
z-score = 0
negative z-score
no activity pattern available
Ratio
-log(p-value)
0
5
10
15
20
25
30
35
Threshold
Th1 and Th2 Activation Pathway
Th1 Pathway
Th2 Pathway
Role of Macrophages, Fibroblasts and Endothelial Cells in Rheumatoid Arthritis
Ovarian Cancer Signaling
Pancreatic Adenocarcinoma Signaling
T Helper Cell Differentiation
G-Protein Coupled Receptor Signaling
NF-κB Signaling
Molecular Mechanisms of Cancer
Colorectal Cancer Metastasis Signaling
Glioma Signaling
Regulation of the Epithelial-Mesenchymal Transition Pathway
Hepatic Fibrosis / Hepatic Stellate Cell Activation
HER-2 Signaling in Breast Cancer
Glucocorticoid Receptor Signaling
Role of Pattern Recognition Receptors in Recognition of Bacteria and Viruses
Hepatic Cholestasis
Dendritic Cell Maturation
Melanoma Signaling
Non-Small Cell Lung Cancer Signaling
p53 Signaling
Type II Diabetes Mellitus Signaling
PI3K Signaling in B Lymphocytes
PEDF Signaling
IL-12 Signaling and Production in Macrophages
Acute Phase Response Signaling
Altered T Cell and B Cell Signaling in Rheumatoid Arthritis
NF-κB Activation by Viruses
Acute Myeloid Leukemia Signaling
Endometrial Cancer Signaling
iCOS-iCOSL Signaling in T Helper Cells
HMGB1 Signaling
PTEN Signaling
Neuropathic Pain Signaling In Dorsal Horn Neurons
Role of Osteoblasts, Osteoclasts and Chondrocytes in Rheumatoid Arthritis
Prostate Cancer Signaling
IL-4 Signaling
Type I Diabetes Mellitus Signaling
B Cell Receptor Signaling
IL-15 Signaling
IL-3 Signaling

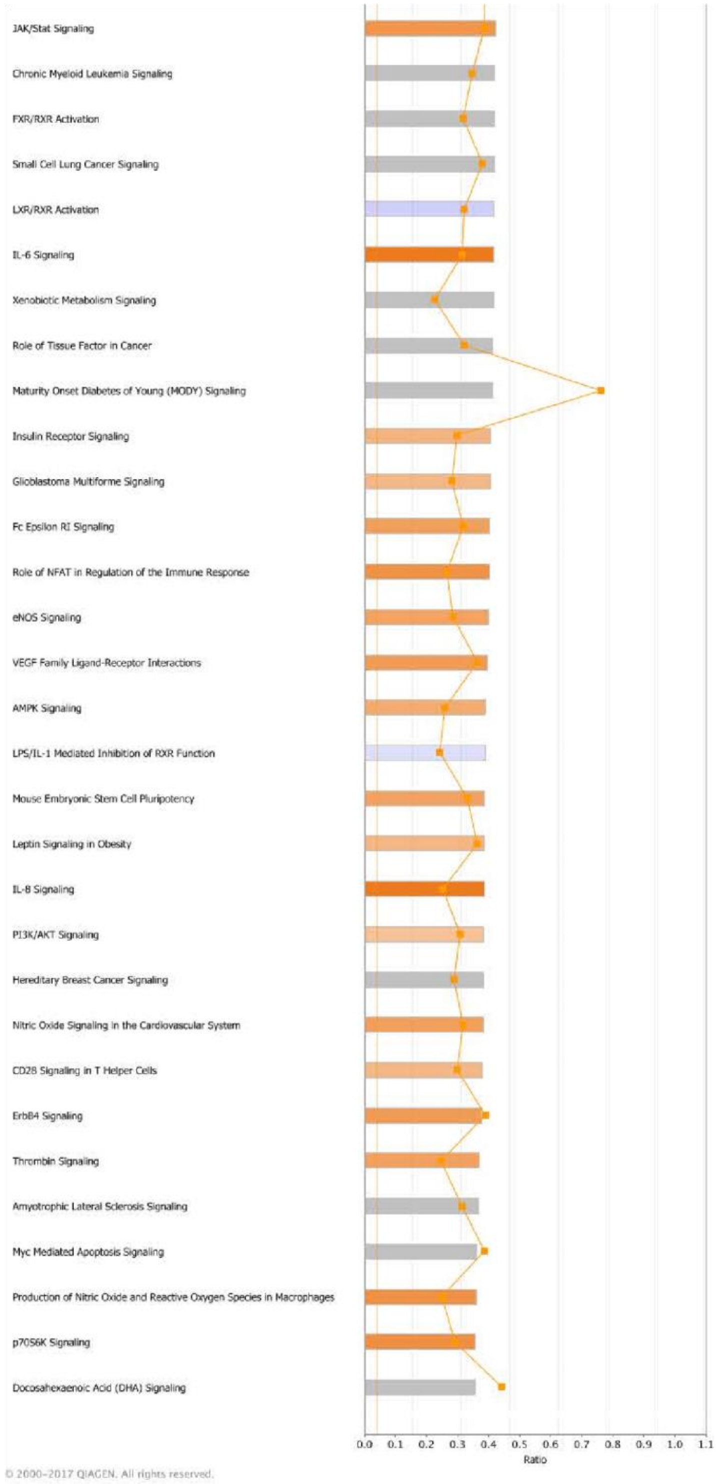

Fig. S7. Canonical pathways across 175 diseases. Ingenuity pathway analysis of GWAS genes belonging to all 175 diseases. The Th1 and Th2 activation pathway was most significant.

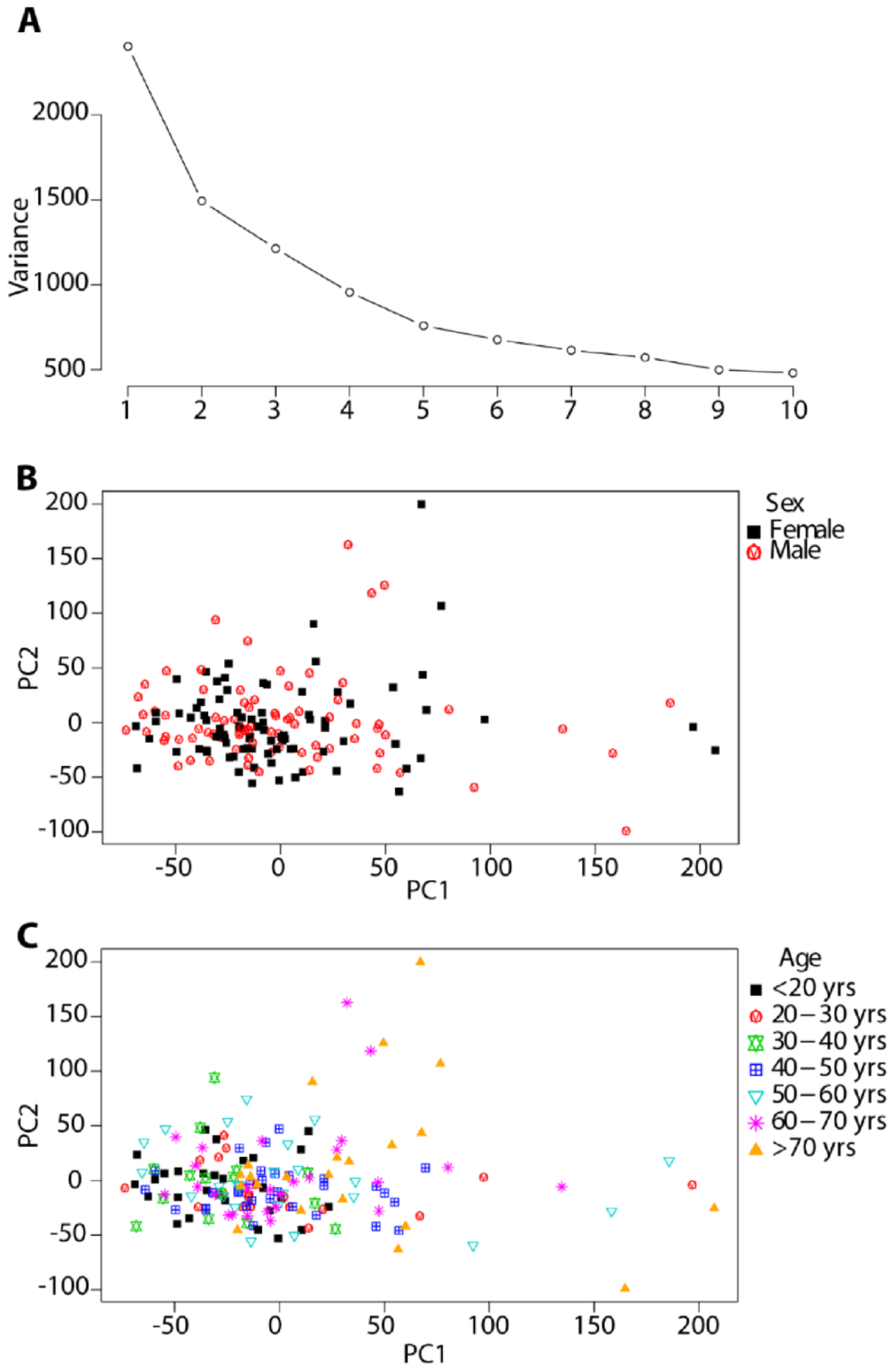

Fig. S8. Principal Component Analysis of 166 microarrays (127 patients and 39 healthy controls). (A) Variance distribution over the principal components. First two principal components with data points colored according to subject's sex**. (B)**, and age **(C)**. For the age confounding test, patients were grouped into categories of <20, 20–30, 30–40, 40–50, 50–60, 60–70, and >70 years of age.

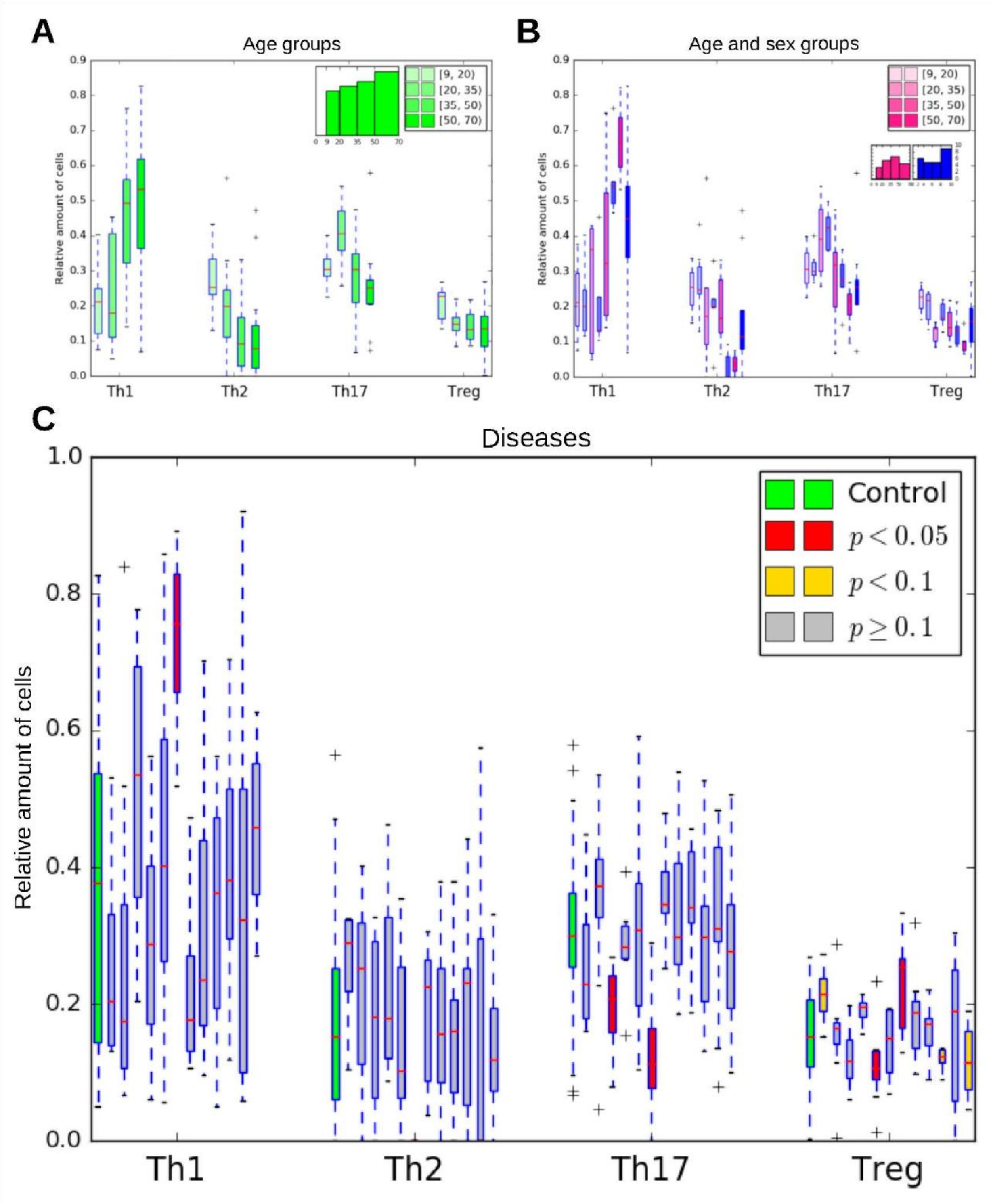

Fig. S9. Predicted percentage from CIBERSORT showed high overlap of T cell composition between different age groups and sexes of patients and controls. (**A**) Box plots of cellular composition in different healthy age groups (darker green means greater age), (**B**) healthy sex and age groups, and (**C**) across the 13 different diseases, and controls. Boxes correspond to quartiles, median is marked within the boxes, whiskers are marked with dashed lines, and outliers are marked with '+' signs.

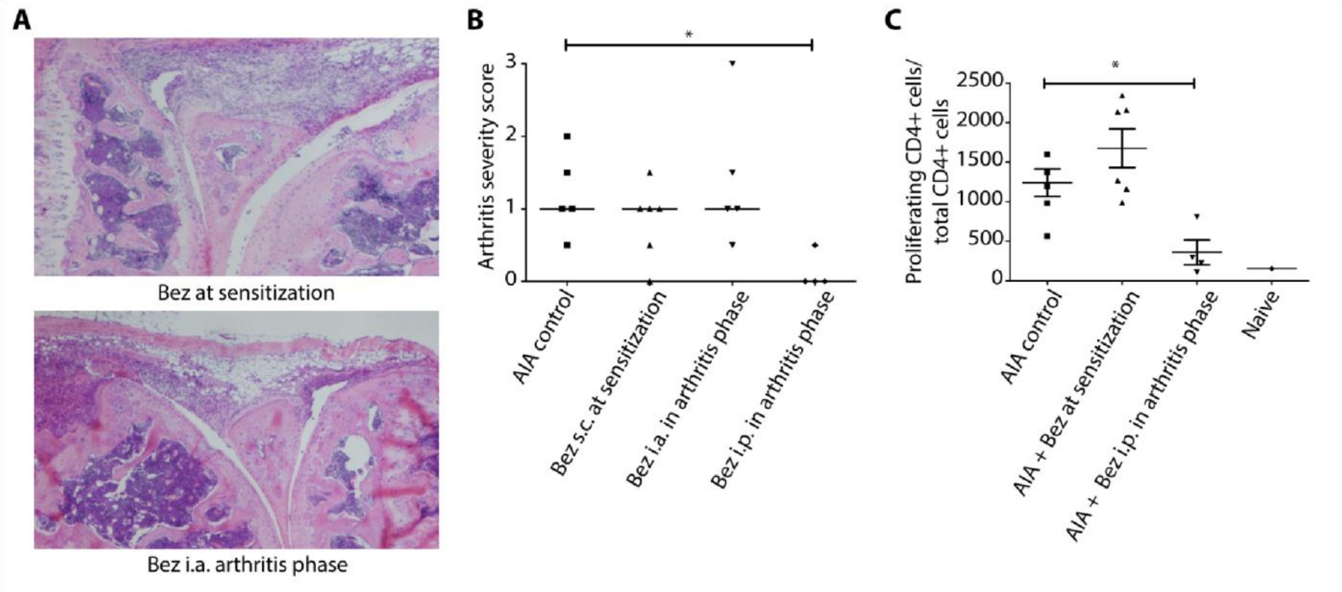

Fig. S10. Bezafibrate protects against antigen-induced arthritis and inhibits antigen-induced CD4+ helper T cell proliferation. Female mice sensitized with mBSA (on days one and seven) were subjected to arthritis via intra-articu-lar injection (day 21) of mBSA and they were mock-treated or subcutaneously (s.c.) treated with bezaf-ibrate at sensitization (8 mg/kg), intraperitoneally (i.p.) on days 21, 24, and 26 (4 mg/kg), or intra-artic-ularly (i.a.) on day 21 (0.6 mg/ kg). (**A**) Representative joint images from s.c. and i.a. treatment groups (**B**) Arthritis severity assessed by histopathology day 28. (vertical bars indicate median, differences be-tween groups evaluated using the Mann-Whitney U-test, *$p < 0.05$); (**C**) Antigen recall response of CD4+ helper T cells among spleen and lymph node cells isolated from mock-treated mice (AIA con-trol, $n = 5$), mice treated with bezafibrate at sensitization (AIA+Bez at sensitization, $n = 6$), and mice treated with bezafibrate i.p. day 21, 24, and 26 (AIA+Bez i.p in arthritis phase) (n = 4); vertical bars indicate mean ± SEM, differences between groups evaluated using the Mann-Whitney U-test, *$p < 0.05$). For comparison, cell proliferation in a non-immunized (naïve) mice is shown in (C).

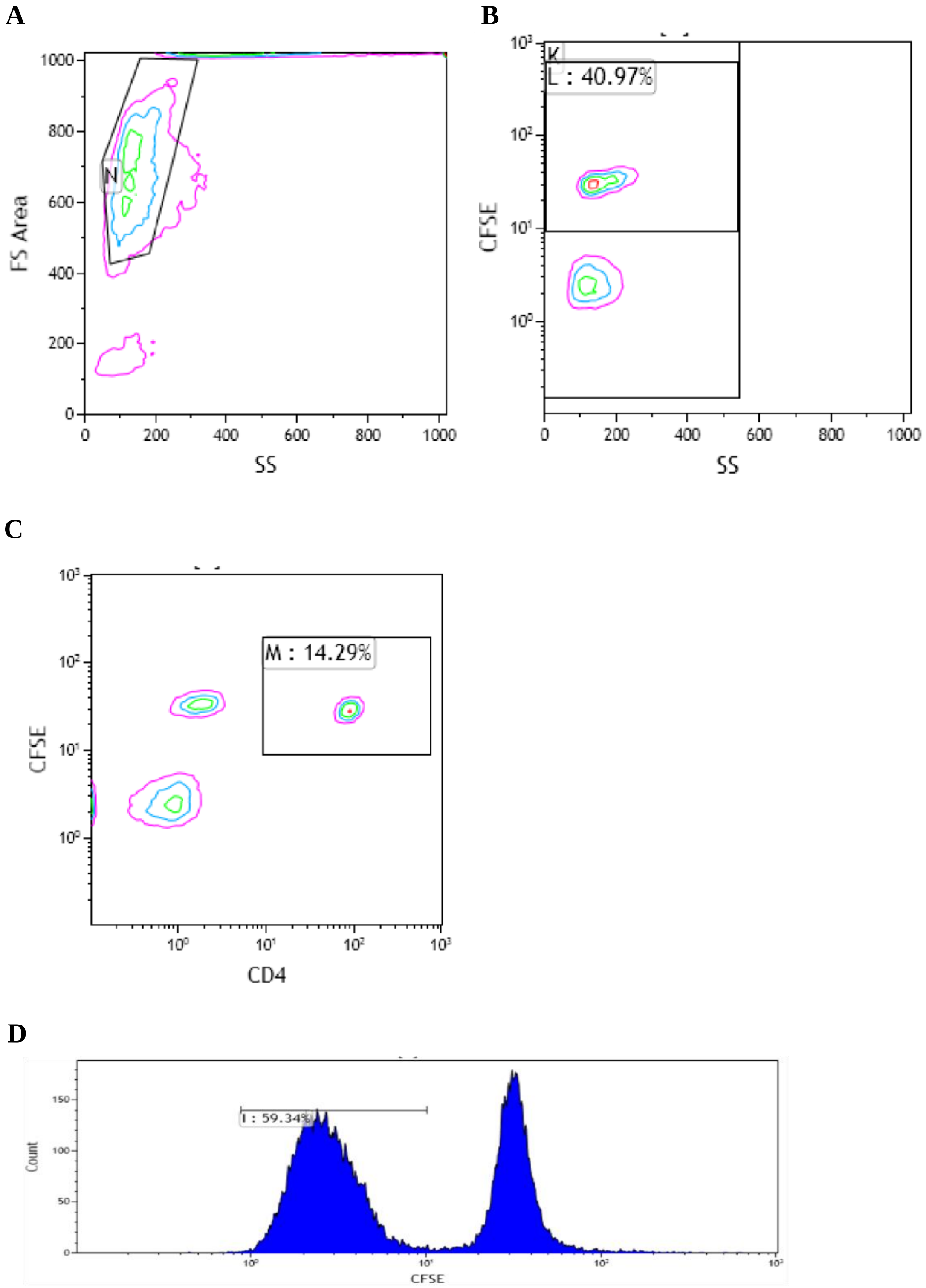

Fig. S11. Gating strategy. For the proliferation analysis, lymphocytes were gated on the basis of the forward and side scatter as shown in **A**. CFSE stained cells were selected from the gated lymphocytes (**B**) and further gated on CD4+ cells (**C**) before analysis of proliferation (**D**).

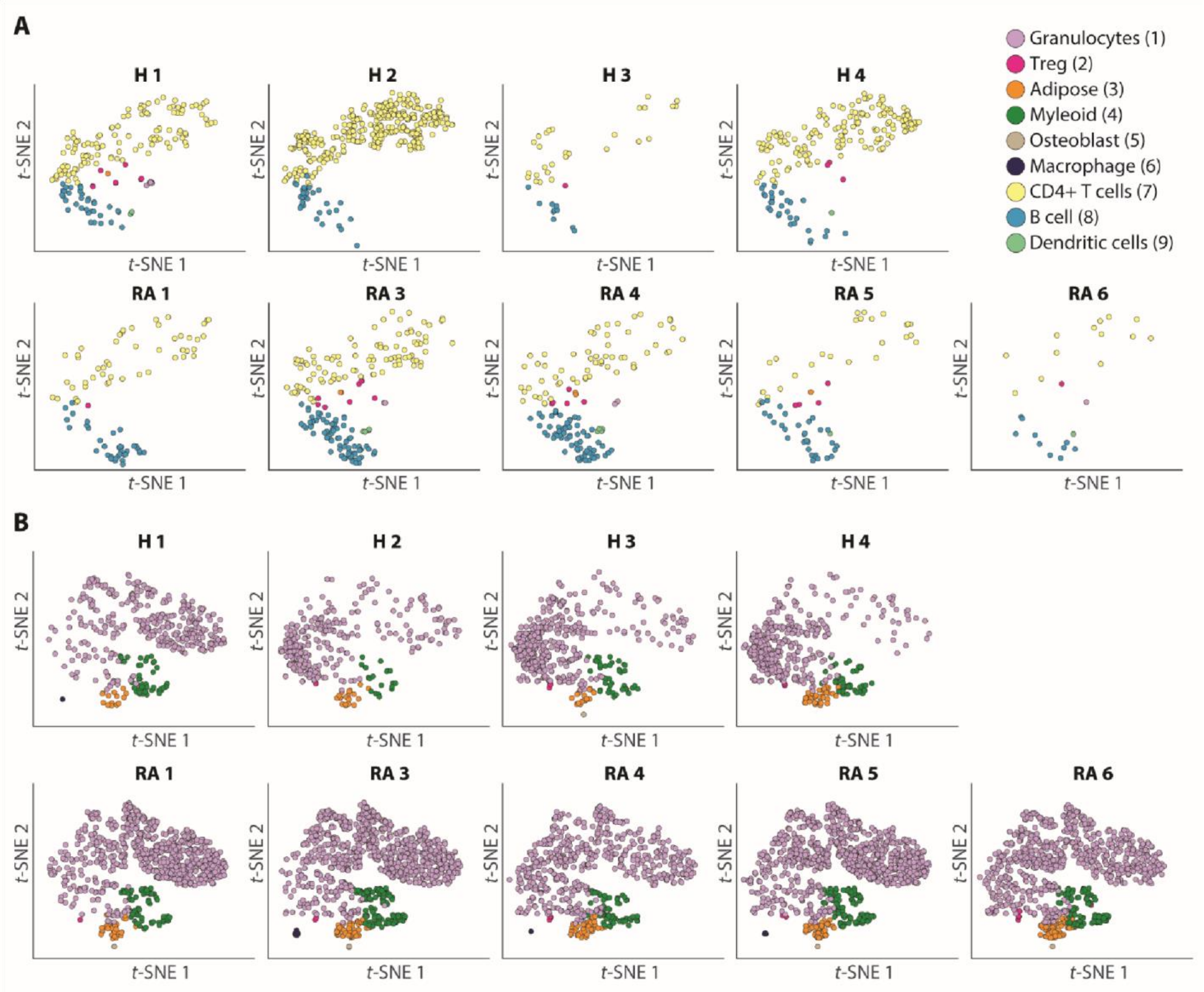

Fig. S12. Cell capture per mouse and condition (*t*-SNE plots). (**A**) t-SNE plot of 1333 healthy and RA lymph nodes cells divided by four healthy mice (H1 to H4) and five RA mice samples (RA1,3 to RA6), colored by RCA clusters, and (**B**) 7086 healthy and RA joint cells divided by four healthy mice (H1 to H4) and five RA mice samples (RA1,3 to RA6). Mouse number 2 had arthritis score of 0.5 (not clearly sick not healthy either) and therefore has been removed from the analyses.

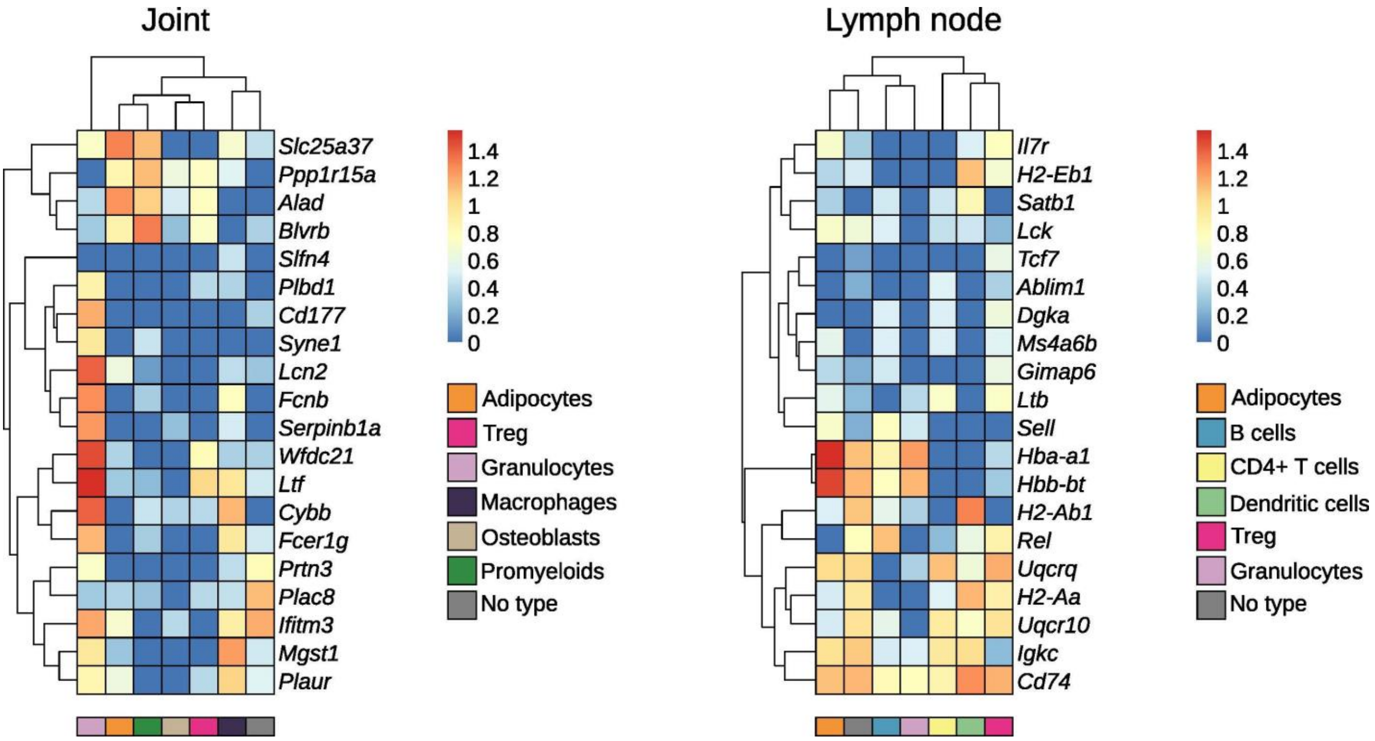

Fig. S13. Mean expression of top cell type specific marker genes for scRNA-seq of cells in joint and lymph node. Heatmap representation of the average expression per cell type of the top 20 most variable genes for RA joint cells (left, n = 7086), and for healthy and AIA lymph node cells (right, n = 1333). We over-lapped the genes from our scRNA-seq data from whole arthritic joints and local lymph nodes with the RCA reference set. For each tissue, we identified the top 100 most present genes, from which we se-lected the top 20 most variable genes.

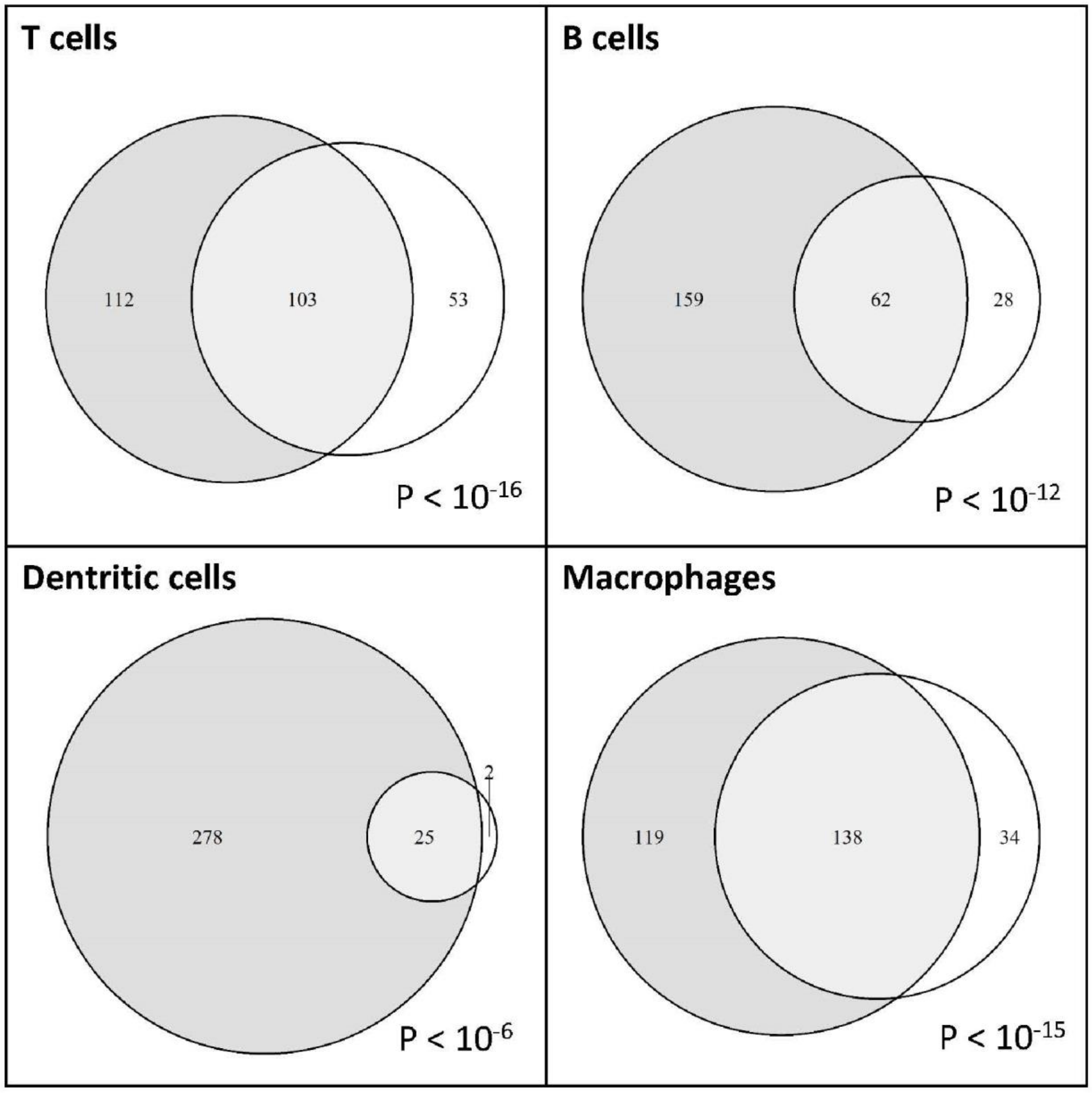

Fig. S14. Comparison of cell type specific pathways from the mouse and human rheumatoid arthritis model. Overlap of rheumatoid arthritis pathways between human and mouse. Mouse pathways (white) and human pathways (grey) were compared for all cell types for which data on both species was available. Statistically, overlap was analyzed with the Fisher exact test (onesided), using the total number of pathways defined in IPA (n = 662) as a background.
