# Supplementary material for: A validated single-cell-based strategy to identify diagnostic and therapeutic targets in complex diseases
Source: Genome Med. 2019 Jul 30;11:47. doi: 10.1186/s13073-019-0657-3 (PMC6664760; doi:10.1186/s13073-019-0657-3)
Supplement: Supplementary file 3 — Contains Tables S1–S4. (PDF 79 kb) [file 13073_2019_657_MOESM3_ESM.pdf]

Supplementary Tables

Supplementary Table 1. Enrichment of rheumatoid arthritis GWAS genes in deregulated genes in different cell types in sick joint samples.

**Cell type**	***p* value**	**OR**	**DEG and GWAS**	**DEG, non-GWAS**	**Non-DEG, GWAS**	**Non-DEG, non-GWAS**
T regulatory cells	0.077	2.27	5	366	164	27194
Adipocytes	8.40 x 10e-7	2.39	53	4507	116	23601
Promyeloids	2.07 x10^-8	2.66	57	4531	112	23698
Granulocytes	5.70 x10^-7	2.39	56	4853	113	23404
Macrophage	0.075	1.58	19	2055	150	25660
Osteoblasts	0.25	2.12	2	155	167	27399

OR, Odds ratio; DEG, differentially expressed genes; GWAS, genome-wide association studies.

Supplementary Table 2. Enrichment of rheumatoid arthritis GWAS genes in different cell types in sick lymph node samples.

Cell type	*p* value	OR	DEG and GWAS	DEG, non-GWAS	Non-DEG, GWAS	Non-DEG, non-GWAS
B cells	0.045	2.12	9	714	160	26933
CD4+ T cells	0.004	2.38	15	1089	154	26605
T-regulatory cells	1		0	0	169	27546
Adipocytes	1	0	0	7	169	27539
Dendritic cells	0.015	11.36	2	29	167	27519
Granulocytes	1	0	0	39	169	27510

OR, odds ratio; DEG, differentially expressed genes; GWAS, genome-wide association studies.

Supplementary Table 3. List of GWAS diseases with ICD-10-CM codes and chapters.

ICD-10-CM chapter name	Number of diseases in chapter (*n*)	ICD-10-CM chapter in Latin numerals
Certain infectious and parasitic diseases	11	I
Neoplasms	37	II
Diseases of the blood and blood-forming organs and certain disorders involving the immune mechanism	3	III
Endocrine, nutritional and metabolic diseases	9	IV
Mental and behavioral disorders	19	V
Diseases of the nervous system	16	VI
Diseases of the eye and adnexa	5	VII
Diseases of the ear and mastoid process	1	VIII
Diseases of the circulatory system	17	IX
Diseases of the respiratory system	6	X
Diseases of the digestive system	14	XI
Diseases of the skin and subcutaneous tissue	8	XII
Diseases of the musculoskeletal system and connective tissue	15	XIII

Diseases of the genitourinary system	8	XIV
Pregnancy, childbirth and the puerperium	1	XV
Certain conditions originating in the perinatal period	1	XVI
Congenital malformations, deformations and chromosomal abnormalities	4	XVII
Symptoms, signs and abnormal clinical and laboratory findings, not elsewhere classified	2	XVIII
Injury, poisoning and certain other consequences of external causes	1	XIX
External causes of morbidity and mortality	0	XX
Factors influencing health status and contact with health services	2	XXI

ICD-10-CM, International classification of diseases, Tenth revision, clinical modification.

Supplementary Table 4. Cellular interaction and association statistics for 45 human cell-types based on manual curation, ENCODE. The columns contain; cell type names, the number of cell-types identified as interaction partners using manual curation, and the number of diseases that were significantly associated with the cell type based on the epigenetic marker disease association score.

cellType	Number of associated diseases (n)	Number of cell type interactions (n)
Astrocyte	77	17
Astrocyte of the cerebellum	69	17
Astrocyte of the spinal cord	69	17
B cell	95	44
Brain microvascular endothelial	67	18
Bronchial epithelial cell	103	16
Cardiac fibroblast	78	18
Cardiac muscle cell	97	16
CD14-positive monocyte	111	44
CD4-positive helper T cell	100	44
CD4-positive, alpha-beta memory	76	44
CD4-positive, CD25-positive, al-	95	44
CD8-positive, alpha-beta memory	45	44
CD8-positive, alpha-beta T cell	93	44
Choroid plexus epithelial cell	72	17
Epithelial cell of esophagus	71	14
Epithelial cell of prostate	83	14
Epithelial cell of proximal tubule	72	14
Fibroblast of arm	112	16
Fibroblast of dermis	87	23
Fibroblast of gingiva	73	13
Fibroblast of lung	104	15
Fibroblast of mammary gland	70	14
Fibroblast of pedal digit skin	75	16
Fibroblast of pulmonary artery	68	13
Fibroblast of skin of abdomen	70	17
Fibroblast of the aortic adventitia	75	14
Fibroblast of upper leg skin	67	16
Foreskin fibroblast	109	16
Foreskin keratinocyte	108	18
Foreskin melanocyte	94	17
Hepatocyte	94	11
Keratinocyte	107	23
Kidney epithelial cell	100	12
Mammary epithelial cell	96	18
Mononuclear cell	108	44

Natural killer cell	95	44
Neural cell	100	40
Neuron	97	39
Neutrophil	121	44
Osteoblast	107	14
Retinal pigment epithelial cell	70	15
Skeletal muscle cell	70	14
Smooth muscle cell	98	22
T-cell	104	44
