# Supplementary material for: A validated single-cell-based strategy to identify diagnostic and therapeutic targets in complex diseases
Source: Genome Med. 2019 Jul 30;11:47. doi: 10.1186/s13073-019-0657-3 (PMC6664760; doi:10.1186/s13073-019-0657-3)
Supplement: Supplementary file 4 — Random walk betweenness centrality of cell types for validation of MCDM in joint tissue. Contains supplementary table describing ligand-receptor based centrality analyses. (PDF 115 kb) [file 13073_2019_657_MOESM4_ESM.pdf]

Additional file 4. Random walk betweenness centrality of cell types for validation of MCDM in joint tissue. Cell-cell interactions for the validation MCDM were predicted from cell-specific scRNA-seq DEGs by using a publicly available mouse ortholog ligand-receptor pairs database (Methods). Weights were based on the number of cells of each cell type and its number of predicted upstream regulators.

cell	rw_score_ligand_receptor
gran	0,834270517
adipo	0,095489052
myelo	0,08753179
macro	0,009663604
treg	0,00128887
osteo	0,001205998
