# Supplementary material for: A validated single-cell-based strategy to identify diagnostic and therapeutic targets in complex diseases
Source: Genome Med. 2019 Jul 30;11:47. doi: 10.1186/s13073-019-0657-3 (PMC6664760; doi:10.1186/s13073-019-0657-3)
Supplement: Supplementary file 5 — Models of 174 diseases based on epigenetic marker enrichments in GWAS genes (excluding rheumatoid arthritis presented in the main text). Compressed folder (ZIP), which can be found as DataS2.zip at: https://figshare.com/articles/DataS2_zip/7976552. (DOCX 14 kb) [file 13073_2019_657_MOESM5_ESM.docx]

**Additional file 5**: Models of 174 diseases based on epigenetic marker enrichments in GWAS genes (excluding rheumatoid arthritis presented in the main text). Compressed folder (ZIP), which can be found as dataS2.zip at <https://figshare.com/articles/DataS2_zip/7976552>.
